# Modulation of Donepezil Release Through Hydrophobic Ion- Pairing with Oleic Acid in Liquid Crystalline Nanodispersions for Transdermal Delivery

**DOI:** 10.1021/acsomega.6c04903

**Published:** 2026-07-17

**Authors:** Priscila Pires da Silva, Victor Cavalcanti Marques, Lorena Kelly Santiago Ramos, José Dias de Souza Filho, Renato Ferreira de Freitas, Marina Guimarães Carvalho Machado, Marcus Vinícius Cangussu Cardoso, Flávia de Souza Marques, Paula Melo de Abreu Vieira, Leonardo da Silva Araújo, Jaqueline dos Santos Soares, Jacqueline de Souza, André Luís Morais Ruela

**Affiliations:** † Graduate Program of Pharmaceutical Sciences, School of Pharmacy, 28115 Federal University of Ouro Preto, UFOP, Ouro Preto 35400-000, Minas Gerais, Brazil; ‡ Graduate Program in Biotechnology, Institute of Exact and Biological Sciences, Federal University of Ouro Preto, UFOP, Ouro Preto 35400-000, Minas Gerais, Brazil; § Graduate Program in Biological Sciences, Institute of Exact and Biological Sciences, Federal University of Ouro Preto, UFOP, Ouro Preto 35400-000, Minas Gerais, Brazil; ∥ Department of Physics, Institute of Exact and Biological Sciences, Federal University of Ouro Preto, UFOP, Ouro Preto 35400-000, Minas Gerais, Brazil

## Abstract

Precursor liquid crystals based on unsaturated monoglycerides, with or without oleic acid (OA), exhibited anisotropy characteristic of an inverse hexagonal phase. Donepezil-loaded liquid crystalline nanodispersions (LCNs) were produced by sonication and exhibited encapsulation efficiencies of 91.3–97.9%, with the OA-containing formulation (LCN-2) displaying a smaller particle size (183.0 ± 1.5 nm), lower polydispersity (0.183 ± 0.006), and improved colloidal stability over 90 days. NMR (^1^H NMR, 1D NOESY/ROESY and DOSY), molecular docking, and molecular dynamics simulations consistently demonstrated hydrophobic ion-pair formation between donepezil and oleic acid. These molecular interactions reduced drug mobility within the lipid domains, resulting in anomalous release behavior (*n* = 0.79), more sustained in vitro release, and an approximately 45% higher steady-state skin flux than the control donepezil solution. Overall, hydrophobic ion-pair formation with oleic acid represents an effective strategy to modulate drug release and enhance the transdermal performance of liquid crystalline nanodispersions for donepezil delivery.

## Graphical abstract

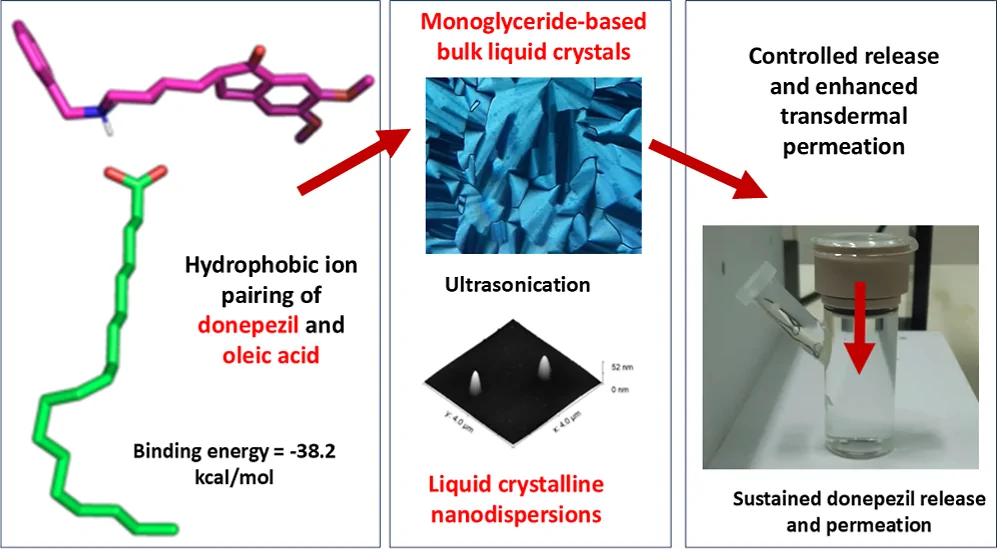

## 1 Introduction

Alzheimer’s disease is a progressive neurodegenerative disorder of the central nervous system, and current treatments rely primarily on acetylcholinesterase inhibitors. Donepezil is a reversible inhibitor that binds to the anionic site of the enzyme, reducing its activity and increasing acetylcholine levels in the brain.^1,2^ Although donepezil hydrochloride is mainly administered orally and widely prescribed, its use is associated with gastrointestinal and cardiovascular side effects. Chemically, donepezil hydrochloride is a white to off-white crystalline powder (mp 224 °C), highly water-soluble, and exhibits basic properties (molecular weight as free base = 379.2 g/mol, p*K*_a_ = 8.9, log *P* = 4.27, and TPSA = 38.8 Å^2^).^3–5^ Although these physicochemical properties make donepezil a promising candidate for transdermal administration, improving its affinity for the lipophilic stratum corneum remains an important challenge, providing the rationale for strategies based on hydrophobic ion-pair formation.^6–8^

Transdermal delivery offers gradual absorption, sustained therapeutic levels, reduced first-pass metabolism, improved adherence, and fewer gastrointestinal effects. These features make it particularly attractive for neurodegenerative diseases such as Alzheimer’s (rivastigmine and donepezil) and Parkinson’s disease (rotigotine).^2,9–11^ In 2022, the FDA approved Adlarity, a weekly transdermal patch (5 mg/day) that contains a mixture of donepezil hydrochloride and donepezil base designed to maintain near-constant plasma concentrations comparable to oral dosing.^2,11^ Despite this clinical success, efficient transdermal delivery of donepezil remains dependent on overcoming the barrier function of the stratum corneum. Consequently, considerable pharmaceutical research has focused on developing formulation strategies capable of enhancing skin permeation while maintaining controlled drug release.^12–14^ In this context, lyotropic liquid crystals (LLCs) have attracted considerable attention owing to their tunable mesophases, structural similarity to biological membranes, and ability to modulate drug transport across the skin.^15,16^

LLCs form through hydration of lipid polar headgroups and the subsequent aggregation of their hydrophobic domains, with unsaturated monoglycerides commonly employed as the lipid components.^17–21^ These nanostructured systems can be dispersed in water to form lyotropic liquid crystalline nanodispersions (LCNs), typically via precursor liquid crystalline phases and often in the presence of additional excipients to optimize their physicochemical properties. LCNs may be produced using bottom-up approaches (emulsification, solvent evaporation) or top-down methods (sonication, microfluidization, high-pressure homogenization), while polymers such as poloxamers provide steric stabilization.^20–22^

Incorporation of oleic acid (OA) into monoolein–water systems alters lipid packing and promotes the formation of inverse hexagonal (H_II_) mesophases, which have considerable potential for drug delivery.^18,20,21^ The H_II_ phase consists of densely packed cylindrical micelles arranged in a two-dimensional hexagonal lattice, with polar headgroups oriented inward and hydrophobic chains extending outward.^22^ This mesophase confers high viscosity and physical stability. Importantly, H_II_ mesophases can be processed into nanodispersions (hexosomes) while retaining structural features advantageous for controlled drug release.^21^ OA is particularly attractive because, besides acting as a lipid component of nanocarriers and a well-established skin penetration enhancer, it can also form ionic complexes with basic drugs such as donepezil.^23^ Such intermolecular interactions may increase drug affinity for lipid matrices, thereby facilitating encapsulation while simultaneously influencing drug release and skin permeation. Supporting this concept, hydrophobic ion-pair formation using sodium oleate has previously been shown to enhance the incorporation of polymyxin B into lipid nanocarriers while providing modulation of the drug release profile.^12^ Likewise, hydrophobic ion pairing between leuprolide and sodium oleate significantly reduced the initial burst release after encapsulation into biodegradable PLGA microspheres.^13^ Nanoemulsions loaded with the lycobetaine–OA ionic complex demonstrated sustained in vitro release driven by ion-pair formation and significantly modulated pharmacokinetics in rats after intravenous administration.^24^ Furthermore, ion-pair formation between donepezil and embonic acid has previously been reported for the development of a long-acting intramuscular formulation, where the hydrophobic complex modulated the drug release profile, sustained plasma donepezil concentrations, and improved pharmacodynamic activity in rats.^25^

Considerable research has been devoted to the development of transdermal delivery systems for donepezil. Both active and passive strategies have been investigated to overcome the barrier properties of the stratum corneum. Active approaches include physical enhancement techniques such as microneedle arrays^26–28^ and iontophoresis,^29–31^ whereas passive strategies have focused on chemical penetration enhancers^6^ and advanced drug delivery systems, including polymeric films,^32^ nanostructured lipid carriers,^7^ nanofibers,^33^ and ionic liquids.^23^ For instance, nanostructured lipid carriers composed of stearic acid and OA have been developed to enhance the transdermal delivery of donepezil,^7^ while donepezil-based ionic liquids prepared with pharmaceutical counterions, including OA, have shown improved permeation in the skin parallel artificial membrane permeability assay (Skin PAMPA).^23^ These studies highlight the potential of lipid-based systems and ionizable counterions to improve the transdermal delivery of donepezil. However, despite these advances, the combination of hydrophobic ion-pair formation with nanostructured liquid crystalline carriers to simultaneously modulate donepezil lipophilicity, enhance skin permeation, and provide controlled drug release has not yet been explored.

Therefore, the development of donepezil-loaded LCNs represents a promising strategy for transdermal delivery in Alzheimer’s disease. This study aimed to prepare and characterize LCNs obtained by sonication, comparing formulations with and without OA to investigate its effects on formulation stability, drug release, and skin permeation. It is hypothesized that ion-pair formation between donepezil and OA governs the supramolecular organization of these systems, acting as a key determinant of drug mobility, release kinetics, and skin permeation. This mechanism may provide a rational strategy for tuning the performance of lipid-based nanocarriers for transdermal delivery.

## 2 Material and Methods

Donepezil hydrochloride (active pharmaceutical ingredient, API; Lot number 04790/2021) was supplied by Laboratório Cristália (Itapira, São Paulo, Brazil). The lipids were monoglycerides of commercial-grade (Myverol 18–92 K, supplied by Kerry Ingredients Co. L., Thailand, 94% of monoglycerides) and OA (Dinâmica Química Contemporânea Ltd.a, Brazil). Methanol HPLC grade was supplied by Merck (Germany). Acetic acid, orthophosphoric acid 85%, sodium hydroxide, sodium acetate trihydrate, sodium chloride, phosphoric acid, and triethylamine were of analytical grade (Dinâmica Química Contemporânea Ltd.a, Brazil). Methacrylic acid (≥99%) was supplied by Sigma-Aldrich (St. Louis, MO, USA). Ultrapure water was obtained using a Milli-Q water purification system.

### 2.1 Donepezil

Donepezil hydrochloride (API) was converted in its free base form to enhance lipid solubility.^19^ Melting points were determined using an M-560 melting point apparatus (Büchi, Switzerland).

### 2.2 NMR Studies

The chemical structures of donepezil and the lipids (unsaturated monoglycerides and OA) were confirmed by proton nuclear magnetic resonance (^1^H NMR) spectroscopy through comparison with reference spectra reported in the literature.^34^ Samples were prepared in 600 μL of deuterated chloroform (CDCl_3_, Sigma-Aldrich, USA). ^1^H NMR and ^13^C NMR spectra were acquired on a Bruker AVANCE III HD 400 MHz high-resolution spectrometer (Bruker BioSpin, Germany). Chemical shifts (δ) are reported in parts per million (ppm) relative to tetramethylsilane (TMS), and coupling constants (*J*) are given in hertz (Hz).

The composition of the lipid excipients used for preparing the formulations was determined by ^1^H NMR. The relative molar contributions of the individual lipid species were calculated by normalizing the integrals of selected signals (bis-allylic protons of polyunsaturated fatty acids, ∼2.8 ppm; olefinic protons, 5.34 ppm; and glyceryl protons of 1- and 2-monoglycerides, ∼4.2 and ∼3.8 ppm, respectively) to the corresponding number of contributing protons, according to the following relationship^34^

1 η∝Ip

where *I* represents the relative integral of the signal and *p* is the number of hydrogen nuclei associated with the resonance. The molar fractions were obtained by dividing each normalized value by the sum of both contributions.

A sample containing donepezil and OA at a 1:2 molar ratio was prepared in 600 μL of deuterated CDCl_3_. Standard ^1^H NMR spectra were acquired using ns = 16, *d*1 = 0.1 s, and sw = 20 ppm. Selective 1D NOESY experiments were performed using the selno pulse program (ns = 254, *d*1 = 2 s, mixing time = 300 ms, sw = 20 ppm) on a spectrometer running TopSpin 4.4.1 software. Selective 1D ROESY experiments were carried out using the selro pulse program (ns = 254, *d*1 = 2 s, sw = 20 ppm) with a continuous-wave (cw) spin-lock and a mixing time of 300 ms.

The degree of ionicity was estimated from ^1^H NMR chemical shifts of samples prepared in deuterated CDCl_3_ using an adapted method.^35^ Based on the observed chemical shift variations, the fraction of ion-paired species was estimated for mixtures of donepezil with model compounds providing either acidic functionality (OA and methacrylic acid) or hydrophobic moieties (unsaturated monoglycerides) at defined molar ratios. The chemical shifts, measured relative to TMS, were used to calculate the percentage of free base (*B* %) according to the following equation

2 $$B\left(\%\right)=\frac{\left(\delta B_{Mix}-\delta BH^{+}\right)\times 100}{\left(\delta B-\delta BH^{+}\right)}$$

3 Degreeofionicity(%)=100−B(%)

where *B* is the chemical shift of free base, BH^+^ is the chemical shift of the fully protonated base (donepezil hydrochloride), and *B*_Mix_ is the chemical shift measured for the prepared mixture.

The following mixtures were prepared: donepezil and OA at molar ratios of 1:2, 1:4, 1:6, and 1:15; donepezil and methacrylic acid at 1:2, 1:4, and 1:8; and donepezil and unsaturated monoglycerides (predominantly C18 monoglycerides) at 1:6, 1:8, and 1:18. This experimental design was intended to distinguish the contribution of acid–base interactions from that of hydrophobic interactions and to evaluate their respective roles in the formation of ion-paired species. Methacrylic acid was selected because it contains a carboxylic acid group but lacks a hydrophobic alkyl chain, whereas the unsaturated monoglycerides possess hydrophobic chains similar to those of OA but do not contain a carboxylic acid group.

^1^H NMR spectra for pure donepezil and OA samples in 600 μL of deuterated CDCl_3_ and for the complex OA/donepezil at a 2:1 molar ratio was obtained using “zgesgp”. The DOSY spectra were recorded with “stegp1s” pulse sequence. Prior to the DOSY measurements, longitudinal (*T*_1_) and transverse (*T*_2_) relaxation times were determined in order to have a better optimization of the diffusion parameters, namely the diffusion time Δ, gradient pulse length δ and relaxation delay (RD). The RD for all relaxation and diffusion measurements were such that RD = 12 > 8*T*_1_ allowing total recovery of magnetizations. The number of scans were (NS = 16) guaranteeing good signal-to-noise ratios for all peaks. The diffusion time period was set Δ = 100 ms ≥ *T*_2_/5, and gradient pulse length of 3 ms for all samples. Gradient strength was varied in the range 2–95% of a maximum amplitude of 53.5 G/cm in 32 linearly spaced gradients. The translational self-diffusion coefficients, *D*_0_, were determined on Bruker built in TopSpin 4.4.1 by fitting Stejskal–Tanner Equation to experimental data

4 I=I0exp(−D0γ2g2δ2(Δ−δ3))

Where γ is the gyromagnetic ratio of proton, *g* is the magnitude of *z*-gradient, δ and Δ are gradient pulse length and diffusion time, respectively. *I* and *I*_0_ are the areas of peaks at a particular gradient strength, *g*, and zero gradient, respectively.

### 2.3 Preparation and Characterization of Donepezil–OA Mixtures

Donepezil base (2.635 mmol) was mixed with OA at donepezil-to-OA molar ratios of 1:1, 1:2, and 1:3 (corresponding to 2.635, 5.270, and 7.905 mmol of OA, respectively). The components were completely dissolved in 1 mL of dichloromethane under gentle stirring. The solvent was then slowly evaporated at room temperature until complete removal, yielding the corresponding bulk mixtures.

The resulting materials were characterized by polarized light microscopy using an optical microscope (Coleman, model P207 LED) equipped with polarizing filters (model N107). Images were acquired at 40× magnification to evaluate optical anisotropy and the possible presence of liquid-crystalline structures.

Raman spectra were acquired using a Standard Microscope Spectroscopy (SMS) system (HORIBA Scientific, Piscataway, NJ, USA) equipped with a 514 nm excitation laser, operated at a laser power of 12 mW at the sample, and a 600 grooves mm^–1^ diffraction grating.

### 2.4 Docking

The coordinates of donepezil and OA were extracted from the Protein Data Bank.^36^ Donepezil coordinates were extracted from its complex with human acetylcholinesterase (PDB ID: 6O4W), while the OA coordinates were taken from its complex with the DegV domain-containing protein MW1315 (PDB ID: 4X9X). Next, hydrogen atoms were added, and the automated docking algorithm DOCKER (available in ORCA,^37^ version 6.1.0) was used to perform the docking calculations at the GFN2-xTB^38^ level in the gas phase.

#### 2.4.1 Molecular Dynamics

Packmol^39^ was used to create a cube of side 25.0 Å, containing one molecule of donepezil (at the center) and six molecules of OA randomly distributed inside the cube.

To simulate the system, the GAFF2 force field^40^ was assigned to the compounds. The GAFF2 parameters were generated using antechamber and parmchk programs. Partial atomic charges were derived using AM1-BCC methodology,^41^ and the system was solvated in a cubic box of chloroform solvent extending at least 10 Å from the complex.

The system was relaxed by two successive energy minimizations using the sander program. First, the heavy atoms of the solute were kept fixed with a 10.0 kcal/mol.Å^2^ force constant while the hydrogen atoms and solvent were relaxed by 1000 steps of steepest descent (SD) before switching to the conjugate gradient (CG) algorithm for the remaining 9000 steps. Next, all atoms were relaxed by another 10000 steps (1000 SD and 9000 CG).

Starting from the minimized system, the complex was equilibrated by three successive steps. First, it was gradually heated from 0 to 300 K during 400 ps with a restraint force constant of 10.0 kcal/mol.Å^2^ applied to heavy atoms of the solute under NVT conditions. Next, a 200 ps MD simulation with a 10.0 kcal/mol.Å^2^ restraint on the solute heavy atoms was carried out at a pressure of 1 atm and 300 K to equilibrate the density using Berendsen’s barostat.^42^ Finally, the system was equilibrated through another 200 ps at 300 K and 1 atm without restraints. The temperature was kept fixed at 300 K using a Langevin thermostat^43^ with a collision frequency of 2 ps^–1^.

After the equilibration phase, we ran 10 MD simulations of 20 ns (200 ns in total) with a target temperature of 300 K and pressure of 1 atm using the AMBER pmemd program.^44^ The SHAKE algorithm^45^ was employed to constrain all hydrogen atoms and the time step was set to 2.0 fs. Particle mesh Ewald (PME)^44^ method was used to treat the long-range electrostatic interactions with a default cutoff of 8.0 Å.

### 2.5 Preparation of Precursor Liquid Crystals and Production of LCNs by Sonication

The aqueous phase (AP) consisted of a phosphate buffer solution (5 mM, pH 7.2) containing Poloxamer 407 (0.5% w/w), methylparaben (0.05% w/w), and propylparaben (0.1% w/w). A mixture of Myverol 18–92 K (0.5 g) and AP (8.8 mL) prior to sonication was designated as the LCN-1 precursor. The OA-containing precursor (LCN-2) was prepared using Myverol 18–92 K (0.4 g), AP (8.8 mL), and OA (0.1 g). Donepezil base (0.075 g) was dissolved in the oil phase of both formulations during the heating step. In both precursor liquid crystalline systems, the monoglycerides were melted at 45 °C in the presence of butylated hydroxytoluene (BHT, 0.02% w/w). The resulting precursor systems were kept at room temperature, protected from light, and allowed to equilibrate for 24 h. Subsequently, the precursor systems were sonicated in an ice bath for 20 min in pulsed mode (10 s on/3 s off) at 80% amplitude using a Sonics VCX-130 Vibra-Cell Ultrasonic Processor (Sonics & Materials, Inc., Newtown, CT, USA) equipped with a titanium probe to produce the LCNs. After sonication, the formulations were designated as LCN-1 (without OA) and LCN-2 (with OA). The final donepezil concentration in the LCNs was 8.02 mg/mL (0.8% w/v). Formulations without drug were prepared and designated as PLCN-1 (without OA) and PLCN-2 (with OA).

### 2.6 Characterizations

#### 2.6.1 Precursor Liquid Crystals

After a 24 h equilibration period with the AP, the precursor systems were sampled and analyzed by polarized light microscopy using an optical microscope (Coleman, model P207 LED) equipped with polarizing filters (model N107) to identify the liquid crystalline phases. Observations were performed at 40× magnification.

#### 2.6.2 LCNs

A preliminary assessment of physical stability was performed by centrifugation at 15,000*g* for 30 min at 25 °C (MicroCen centrifuge, Herolab GmbH, Germany). After centrifugation, the samples were visually inspected for phase separation, sedimentation, or other physical alterations.

Particle size distribution was determined by dynamic light scattering (DLS) using a Zetasizer Nano ZS (Malvern Instruments, Worcestershire, UK) equipped with a 633 nm laser and a fixed scattering angle of 173°. The hydrodynamic diameter (Z-average diameter, HD) and polydispersity index (PDI) were measured for LCNs diluted at 1:200 (v/v) in ultrapure water. The HD was calculated using the cumulant analysis method, which assumes a monomodal Gaussian distribution of the primary particle population and provides the PDI value. The zeta potential (ZP) of LCNs, diluted 1:200 (v/v) in a 0.68 mM sodium chloride solution, was determined by laser Doppler anemometry (LDA) using folded capillary cells (DTS1070, Malvern Instruments, UK) to assess surface charge under low ionic strength conditions. The formulations were stored at two different temperatures, room temperature (20–25 °C) and refrigerated conditions (5–10 °C), and were analyzed every 15 days for up to 90 days to evaluate their physical stability. All measurements of particle size distribution and ZP were performed in triplicate at a controlled temperature of 25 °C (*n* = 3).

The pH of the LCNs with and without donepezil was determined by preparing a dispersion of the formulations in deionized water at 10% w/v.

Rheological measurements of LCNs, with or without donepezil, were performed 24 h after preparation to characterize their flow behavior using a Brookfield LVDV-III Ultra rheometer (Middleboro, MA, USA). Measurements at 25 °C were conducted at progressively increasing rotation speeds to obtain ascending curves (150–200 rpm, in 12.5 rpm increments) with a CP-40 spindle. For all samples, the measurements were subsequently repeated in the reverse direction to generate descending curves.

### 2.7 Evaluation of Donepezil Loading

The encapsulation efficiency (EE) and drug loading (DL) of donepezil were determined by ultrafiltration using Amicon Ultra centrifugal filter devices (50 kDa molecular weight cutoff, Millipore, Darmstadt, Germany). For this purpose, 0.4 mL of donepezil-loaded LCNs was added to the filter unit, previously prewashed with ultrapurified water, and centrifuged at 1750*g* for 30 min at 25 °C. Donepezil was quantified in the total LCN dispersion and in the ultrafiltrate, after separating the nanoparticles from the AP, by the HPLC.

EE was calculated according to

5 EE(%)=(Totalamountofdonepezil−amountoffreedrug/Totalamountofdonepezil)×100

where amount of free drug corresponds to the amount of nonencapsulated drug recovered in the ultrafiltrate, and Total amount of donepezil represents the total drug content in the LCN dispersion. The DL expresses the amount of drug effectively encapsulated within the nanoparticles (total amount of donepezil- amount of free drug) relative to the weight of lipids, defined as the sum of all lipid components present in the oil phase of the LCNs, and was calculated according to

6 DL(%)=Totalamountofdonepezil−amountoffreedrug/Weightoflipids×100

### 2.8 In Vitro Release Studies

In vitro release studies were performed using a vertical Franz-type glass diffusion cell apparatus equipped with a magnetic stirrer and an automatic sampling system (Phoenix, Teledyne Hanson Research, Chatsworth, CA, USA). Each diffusion cell had a receptor volume of 22 mL and an effective diffusion area of 1.33 cm^2^. The system was maintained at 37.0 ± 0.5 °C. The receptor compartment was filled with acetate buffer (pH 4.5) and stirred at 100 rpm, which was selected to ensure sink conditions.^5,19^ A volume of 200 μL of either the donepezil control solution or LCNs was applied to the donor compartment, directly onto highly permeable cellulose dialysis membranes (12 kDa molecular weight cutoff, Merck) mounted between the donor and receptor compartments. Prior to use, the dyalisis membranes were washed with water at 80 °C to remove storage residues. One-milliliter aliquots were automatically withdrawn at 0.5, 1, 2, 3, 4, 6, 9, 12, and 18 h and immediately replaced with an equal volume of fresh receptor phase. All experiments were performed in sextuplicate (*n* = 6).

Donepezil quantification in the receptor phase during the release studies was performed by HPLC, as previously described.

#### 2.8.1 Donepezil Control Solution

To prepare the donepezil control solution, 24.06 mg of donepezil was accurately weighed and initially dissolved in 200 μL of ethanol to ensure complete dissolution. Subsequently, 2.8 mL of sodium acetate buffer (pH 4.5) was added, resulting in a final ethanol concentration of 6.7% (v/v).

### 2.9 Mathematical Modeling

The kinetics of drug release was evaluated by plotting the mean cumulative percentage of drug released as a function of time (*h*). The release data were fitted to the Korsmeyer–Peppas (logarithm of the concentration against logarithm of the time)^46^

7 $$M_{t}/M_{0}=K_{n}t^{n}$$

Where the ratio *M*_*t*_/*M*_*0*_ represents the fractional drug release at time *t* (h), *n* is the release exponent that characterizes the drug release mechanism, and *K*_*n*_ is the Korsmeyer–Peppas rate constant. The correlation coefficient (*r*) was calculated to evaluate the goodness of fit of the model*.*

### 2.10 Skin Absorption

Skin permeation studies were performed using the same experimental setup described for the in vitro release studies and conducted at 37 ± 1 °C. Full-thickness porcine ear skin, comprising the epidermis and part of the dermis, was excised using scalpels. Skin samples were obtained from a local slaughterhouse (Juiz de Fora, MG, Brazil). The freshly excised skin was mounted between the donor and receptor compartments, with the stratum corneum facing the donor side. Donepezil-loaded formulations or the control solution (200 μL) were then applied directly onto the skin in the donor compartment.

The receptor chamber of the diffusion cells was filled with sodium acetate buffer (pH 4.5) and continuously stirred at 300 rpm throughout the experiments. One-milliliter aliquots were withdrawn at 2, 4, 6, 9, 12, 18, 24, 30, 36, and 48 h and immediately replaced with an equal volume of fresh receptor medium. All experiments were performed in sextuplicate (*n* = 6). Donepezil in the receptor phase was quantified by HPLC. The permeation data were fitted to the zero-order model (concentration against time)^46^

8 $$Q_{t}=Q_{0}+K_{0}t$$

where *Q*_*t*_ represents the cumulative amount of drug permeated (μg) at time *t* (h), *Q*_0_ is the initial amount of drug in the donor compartment, and *K*_*0*_ denotes the zero-order rate constant (μg h^–1^). The *r* was calculated to evaluate the goodness of fit of the model.

The steady-state flux (*J*_*ss*_, μg/cm^2^/h) was calculated from the slope of the linear region of the cumulative permeated amount per unit area versus time plot. The lag time (*T*_lag_) was determined by extrapolating this linear region to the *x*-axis. The apparent permeability coefficient (*K*_p_, cm/h) was calculated as

9 Kp=JssC

Where (*C*, μg) is the initial drug concentration in the donor formulation.

After permeation studies (24 and 48 h), whole skin fragments were removed, briefly washed with water, and placed in polyethylene tubes (*n* = 6). Skin homogenates were prepared by homogenizing whole skin fragments with 5 mL of receptor phase. Subsequently, 5 mL of methanol was added, and the samples were homogenized again. The resulting skin homogenates were centrifuged at 15,000*g* for 15 min, and an aliquot of the supernatant was collected for donepezil analysis.

### 2.11 Donepezil Quantification

Chromatographic analyses were performed using a high-performance liquid chromatography (HPLC) system (UltiMate 3000, Thermo Fisher Scientific, Waltham, MA, USA). The analytical conditions were adapted from a previously reported method, with minor modifications.^47^ Separation was carried out on an C18 end-capped column (150.0 × 4.6 mm i.d., 5 μm particle size; Athena C18-WP, CNW Technologies), maintained at 30 °C. The mobile phase was used in isocratic mode at a flow rate of 1.0 mL min^–1^, with an injection volume of 20 μL. UV detection was performed at 268 nm. The mobile phase consisted of 0.1% (v/v) triethylamine adjusted to pH 3.0 with acetic acid and methanol in a ratio of 55:45 (v/v). Prior to analysis, samples were filtered through a 0.22 μm hydrophilic polyvinylidene fluoride (PVDF) syringe filter. Under these conditions, donepezil eluted at approximately 6.1 min, and the total run time was 10 min.

Method validation was conducted in accordance with the International Council for Harmonisation (ICH) guidelines, confirming its suitability of the method for donepezil quantification.^48^ The method showed linearity over the concentration range of 0.13–50 μg mL^–1^ for the determination of donepezil in the receptor medium (sodium acetate buffer, pH 4.5) used in in vitro drug release and skin absorption studies. In addition, as previously established by the research group,^5,19^ linearity was observed in the range of 0.5–150 μg mL^–1^ for donepezil quantification in a 0.1% triethylamine solution (pH 3.0, adjusted with acetic acid), which was employed for drug loading (DL) and encapsulation efficiency (EE) analyses.

### 2.12 Preliminary Cytotoxicity

#### 2.12.1 Cell Culture Using MRC-5 Cell Line

MRC-5 human fetal lung fibroblast cells were cultured in RPMI 1640 medium supplemented with 10% fetal bovine serum (FBS). Cells were maintained at 37 °C in a 5% CO_2_ atmosphere until reaching 80–100% confluence.

#### 2.12.2 MTT Assay

The preliminary cytotoxicity of donepezil and LCNs was evaluated using 3-(4,5-dimethylthiazol-2-yl)-2,5-diphenyltetrazolium bromide (MTT). MRC-5 cells (5 × 10^5^ cells/well) were seeded in 96-well plates and incubated at 37 °C in a 5% CO_2_ atmosphere for 24 h. Subsequently, cells were treated with donepezil (0.38–57.0 μg/mL), LCNs loaded with donepezil, and blank LCNs (without drug). LCNs were used in amounts equivalent to the pure drug. Cells treated with 0.5% dimethyl sulfoxide (DMSO) served as the vehicle control to confirm the absence of cytotoxicity at this concentration.

After treatment, MTT solution (1 mg/mL) was added to each well and incubated for 4 h. Formazan crystals were dissolved in DMSO for 20 min at room temperature, and absorbance was measured at 490 nm using a Tecan Infinite 200 PRO microplate reader (Tecan Group Ltd., Männedorf, Switzerland). Cell viability percentages were calculated for donepezil and LCN treatments. Data are presented as mean ± standard deviation (SD) (*n* = 5).

### 2.13 Statistical Analysis

Data are expressed as mean ± SD. Apparent viscosity and flow curve data were analyzed using one-way analysis of variance (ANOVA), followed by Tukey’s post hoc test. In vitro skin permeation parameters, including drug flux adjusted to zero-order kinetics and lag time, were analyzed using an unpaired Student’s *t*-test. Cell viability data were analyzed using one-way ANOVA, followed by Bonferroni’s post hoc test.

## 3 Results and Discussion

### 3.1 Preformulation

The donepezil free base was characterized by NMR spectroscopy,^5^ confirming its identity (Supporting Information, Figures S1 and S2). The donepezil free base exhibited a melting point of 94 °C, markedly lower than that of donepezil hydrochloride (220–225 °C). This lower melting point is consistent with its higher lipid solubility, as previously reported.^19^

In both lipid samples analyzed by ^1^H NMR, OA and Myverol 18–92 K, signals attributable to polyunsaturated fatty acids were observed at approximately 2.7–2.8 ppm. This information is relevant because lipid composition strongly influences the formation of liquid-crystalline mesophases.^18,22^ For Myverol 18–92 K (Supporting Information, Figure S5), the relative contribution of polyunsaturated chains was estimated from the ratio between the bis-allylic protons (2.78 ppm), which are diagnostic of polyunsaturated fatty acids, and the total olefinic protons (5.34 ppm), which reflect the total number of double bonds. Considering that *p* is the number of hydrogen nuclei contributing to each resonance, this value was 4 for the signal at 2.78 ppm, consistent with ω-6 unsaturated chains, as supported by the absence of the ω-3 acyl signal at 0.97 ppm. On this basis, approximately 44.5% of the double bonds in the sample were associated with polyunsaturated chains, whereas 55.5% corresponded to nonbis-allylic unsaturations. These values should be interpreted as relative and semiquantitative.^34^ In the ^13^C NMR spectrum of Myverol 18–92 K (Supporting Information, Figure S6), prominent olefinic resonances were detected at 130.44, 130.22, 128.27, and 128.08 ppm.^49^ The signals at 130.44 and 130.22 ppm are consistent with the vinylic carbons of C18:1 (*cis*-9) fatty acids, whereas those at 128.27 and 128.08 ppm are compatible with diunsaturated C18:2 species. A minor olefinic signal was also observed at 129.91 ppm. These assignments are supported by the characteristic downfield shifts typically observed for double bonds in long-chain fatty acids; however, because of the substantial signal overlap in this region, they should be interpreted in conjunction with, and are further corroborated by, the characteristic olefinic resonances observed in the ^1^H NMR spectra.

Similarly, the contribution of polyunsaturated chains in OA (Supporting Information, Figure S3) was estimated from the ratio between the bis-allylic protons (2.75 ppm) and the total olefinic protons (5.38 ppm). Approximately 11.3% of the double bonds were associated with polyunsaturated chains, whereas 88.7% corresponded to nonbis-allylic unsaturations. The presence of polyunsaturated fatty acids was further supported by two additional low-intensity signals (δ = 128.05 and 127.94 ppm) in the ^13^C NMR spectrum of OA, located near the two major olefinic resonances (δ = 130.04 and 129.74 ppm) assigned to OA (C18:1, *cis*-9).^49^ All four signals appeared as positive peaks in the DEPT-135 experiment, consistent with olefinic CH carbons (Supporting Information, Figure S4). Several additional low-intensity resonances observed in the aliphatic region (δ ≈ 31.6, 29.6–29.3, 25.7, and 22.6 ppm) appeared in negative phase in the DEPT-135 spectrum, confirming their assignment to methylene (CH_2_) carbons. These minor signals are consistent with the presence of fatty acids differing slightly in chain length and unsaturation.

Myverol 18–92 K, the primary component of the formulations developed in this study, is a monoglyceride-rich lipid mixture (94%), with ^1^H NMR analysis indicating a predominance of 1-monoglycerides. The ^1^H NMR spectrum exhibited two well-resolved signals attributable to the isomers 1-monoglyceride and 2-monoglyceride, with no spectral overlap (Supporting Information, Figure S5). The resonance at 4.19 ppm, assigned to 1-monoglyceride, and the signal at 3.82 ppm, assigned to 2-monoglyceride, were integrated within the same spectrum, allowing direct comparison of their relative contributions.^34^

The resonance at 4.19 ppm was normalized to a relative integral of 2.0 (two protons), whereas the signal at 3.82 ppm showed a relative integral of 0.28 (four protons). After normalization of the integrals to the respective number of contributing nuclei, the calculated relative molar contributions were 1.00 for 1-monoglyceride and 0.07 for 2-monoglyceride. Based on these values, within the total monoglyceride fraction, 1-monoglyceride represented 93.5% and 2-monoglyceride 6.5%. Glyceryl monooleate (monoolein) is a well-known 1-monoglyceride capable of forming lyotropic liquid crystalline phases, making the ^1^H NMR analysis especially relevant for the present study.^34,50^ However, monoolein is formed by esterification of OA, a monounsaturated fatty acid, whereas our sample (Myverol 18–92 K) contains a relatively high proportion of polyunsaturated fatty acids. This finding indicates that the pool of 1-monoglycerides comprises a more diverse set of unsaturated acyl chains than would be expected for pure monoolein. In the ^13^C NMR spectrum of Myverol 18–92 K (Supporting Information, Figures S6), signals at 70.45, 65.35, and 63.52 ppm were observed, consistent with 1-monoacyl esters.^51^ No signal attributable to 2-monoacyl esters was detected, most likely due to their low proportion in the sample.

The acid–base nature of the interaction between donepezil and OA was evaluated using methacrylic acid and unsaturated monoglycerides (Myverol 18–92 K) as controls (Supporting Information, Figures S7–S12 and Table S1). Methacrylic acid contains a carboxylic acid moiety but lacks the hydrophobic aliphatic chain present in OA, whereas unsaturated monoglycerides possess a similar hydrophobic aliphatic chain but do not contain a carboxylic acid moiety. The chemical shifts used for the ionicity calculations were determined from the benzylic methylene protons of the *N*-benzyl substituent of donepezil. Both OA and methacrylic acid induced significant ^1^H NMR chemical shift changes in donepezil, corresponding to an estimated protonation level of approximately 68.6–81.4% relative to the hydrochloride salt.^35^ These results indicate a partial and dynamic proton transfer equilibrium rather than complete salt formation, consistent with the low dielectric constant of CDCl_3_ and the nonaqueous nature of the system, as well as with the limited thermodynamic driving force for proton transfer in these systems, as reflected by the estimated Δ*pK*_a_ values (donepezil/OA ≈ −0.8; donepezil/methacrylic acid ≈ +4.5).^5,18^ In contrast, unsaturated monoglycerides, even at high molar excess (up to 18 equiv), produced negligible chemical shift variations (<0.03 ppm). As these lipids retain the oleyl chain but lack a carboxylic acid functionality, the results suggest that the observed NMR perturbations are primarily driven by acid–base interactions, whereas hydrophobic contributions alone do not lead to measurable proton transfer in donepezil under the investigated conditions.

All donepezil–OA bulk mixtures formed oily liquids at room temperature. Polarized light microscopy revealed optical anisotropy at a molar ratio of 1:1, whereas mixtures with molar ratios of 1:2 and 1:3 remained optically isotropic under the same conditions (Supporting Information, Table S2). Intermolecular interactions were further investigated using the nuclear overhauser effect through one-dimensional NOESY experiments, which probe through-space dipolar interactions between nuclei in close spatial proximity. Complementary information was obtained from one-dimensional ROESY experiments, which are particularly suitable for systems in which conventional NOE responses are weak, null, or ambiguous.^52^

Using this combined NOESY/ROESY approach, intermolecular contacts between donepezil and OA in the 1:2 molar mixture were evidenced (Figure 1A). Selective irradiation of the OA methylene protons at 2.3 ppm, corresponding to the methylene group adjacent to the carboxyl moiety, resulted in the appearance of a distinct cross-relaxation signal with opposite phase at 7.36 ppm, assigned to the aromatic protons of the monosubstituted benzene ring of donepezil.

**Figure 1:**
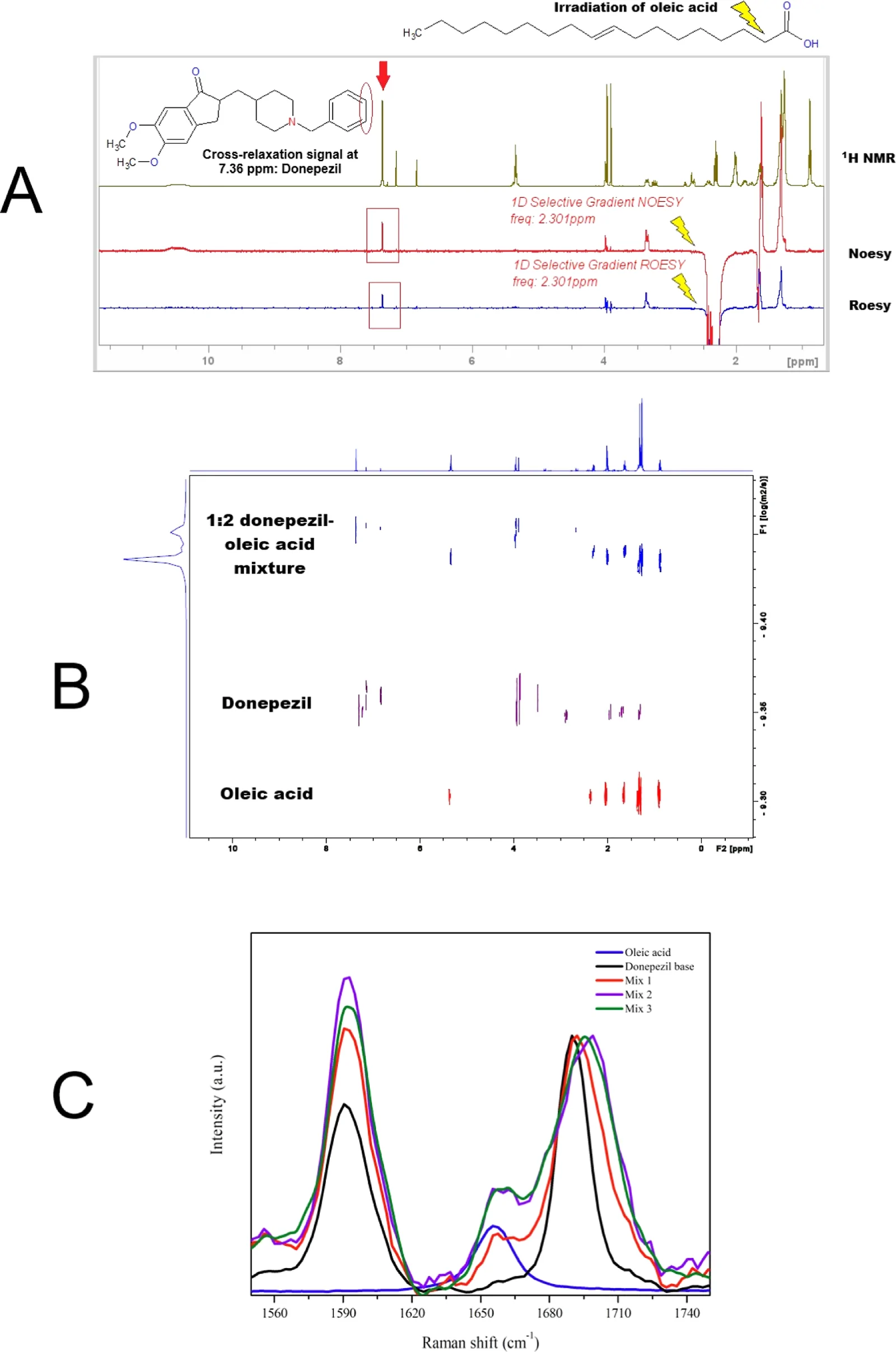
(A) 1D NOESY spectrum (400 MHz, CDCl_3_) of a donepezil and oleic acid mixture at a 1:2 molar ratio, acquired using the selno pulse program with selective irradiation at 2.30 ppm, corresponding to the oleic acid methylene protons adjacent to the carboxyl group. (B) DOSY spectrum (400 MHz, CDCl_3_) showing the diffusion behavior of pure donepezil, pure oleic acid, and the 1:2 mixture, highlighting the convergence to a single diffusion coefficient with the most negative log D value for the mixture. (C) Expanded Raman spectra of donepezil, oleic acid, and mixtures prepared at donepezil-to-oleic acid molar ratios of 1:1 (Mix 1), 1:2 (Mix 2), and 1:3 (Mix 3), acquired using a 514 nm excitation laser, highlighting the 1550–1750 cm^–1^ spectral region.

The DOSY experiment revealed a clear and physically consistent diffusion pattern among the three samples: donepezil alone, OA alone, and a donepezil/OA mixture at a 1:2 molar ratio (Figure 1B). The formation of the ionic pair produced a substantial reduction in the self-diffusion coefficient (*D*_0_). Diffusion analysis showed that the mean self-diffusion coefficient for free OA (∼4.88 × 10^–10^ m^2^·s^–1^) was slightly higher than that of free donepezil (4.45 × 10^–10^ m^2^·s^–1^). Assuming an apparent hydrodynamic radius (a) for a representative spherical model, the ratio a_DON/a_OA ≅ 1.10 suggests that donepezil diffuses with an effective hydrodynamic radius approximately 10% larger than OA under these conditions (CDCl_3_, 15 mM). The *D*_0_ value obtained here is in good agreement with literature data^53^ for oleate in CDCl_3_ (5.75 × 10^–10^ m^2^ s^–1^), supporting the reliability of the measurements. The slightly lower diffusion coefficient observed for OA in this study may be attributed to trace water in the NMR sample, which can promote intermolecular association and thus reduce molecular mobility. When OA and donepezil are combined, the DOSY signals of both species converge to the same diffusion coefficient (3.60 ± 0.06 × 10^–10^ m^2^·s^–1^), indicating that they diffuse together as a single entity. This behavior strongly supports the formation of an associated structure, consistent with an ionic pair.

Remarkably, the donepezil/OA mixture at 1:2 molar ratio presented the most negative log *D* value of all samples, reflecting the slowest diffusion and, therefore, the largest hydrodynamic radius observed in the study. The substantial decrease in diffusion observed for the mixture indicates that the association between protonated donepezil and oleate anions leads to the formation of a supramolecular assembly significantly larger than either molecule alone. The presence of excess OA (2 equiv) favors hydrophobic interactions among oleate chains after ion-pair formation, promoting intermolecular association between these ion pairs and leading to organized aggregates in solution.

Thus, rather than behaving as independent species or as a small ion pair, donepezil and oleate in the 1:2 system diffuse as part of a larger, organized supramolecular entity. The DOSY data therefore provide strong evidence that ion-pair formation triggers higher-order self-assembly driven by hydrophobic interactions of the oleate chains, resulting in a new diffusive identity with an increased hydrodynamic size.

Raman spectroscopy showed a systematic shift of the donepezil C=O stretching band (∼1690 cm^–1^) toward higher wavenumbers upon increasing OA content, accompanied by band broadening (Figure 1C). These changes are consistent with alterations in the electronic environment of the carbonyl group resulting from acid–base interactions between donepezil and OA, in agreement with ion-pair formation inferred from the NMR experiments. The band broadening likely reflects heterogeneous ion-paired microenvironments within the lipid assembly. Outside this region, the Raman profiles remained essentially unchanged, indicating preservation of the molecular structures and confirming that the interaction is noncovalent in nature. A similar spectroscopic behavior has been reported for donepezil incorporated into the acrylic pressure-sensitive adhesive Duro-Tak 87–4098, where Fourier-transform infrared spectroscopy revealed a shift of the carbonyl stretching band from 1682 to 1703 cm^–1^ ^23^. The agreement between these previous observations and the Raman spectral changes observed here further supports the occurrence of molecular interactions involving the carbonyl group of donepezil.

To complement the experimental NMR and Raman spectroscopy findings and provide a molecular-level interpretation of the observed intermolecular interactions, molecular docking and molecular dynamics simulations were performed to investigate the structure and stability of the donepezil–OA complex. Initially, the DOCKER program was employed to predict the most plausible binding poses of the complex. The calculation resulted in 16 poses of the OA-donepezil complex (OA-DP), which were clustered in six unique poses (Figure 2A). The top-scored complexes were **C1** (*E* = −41.3 kcal/mol) and **C2** (*E* = −38.3 kcal/mol), where both form a salt–bridge interaction between the carboxylate of OA and the positively charged nitrogen of the piperidine ring. In the **C1** pose, the long alkyl chain of OA is stretched along the 5,6-dimethoxy-1-indanone moiety of donepezil, making several hydrophobic interactions. This could explain why this pose was slightly more stable than **C2**, where OA is oriented perpendicularly in relation to donepezil.

**Figure 2:**
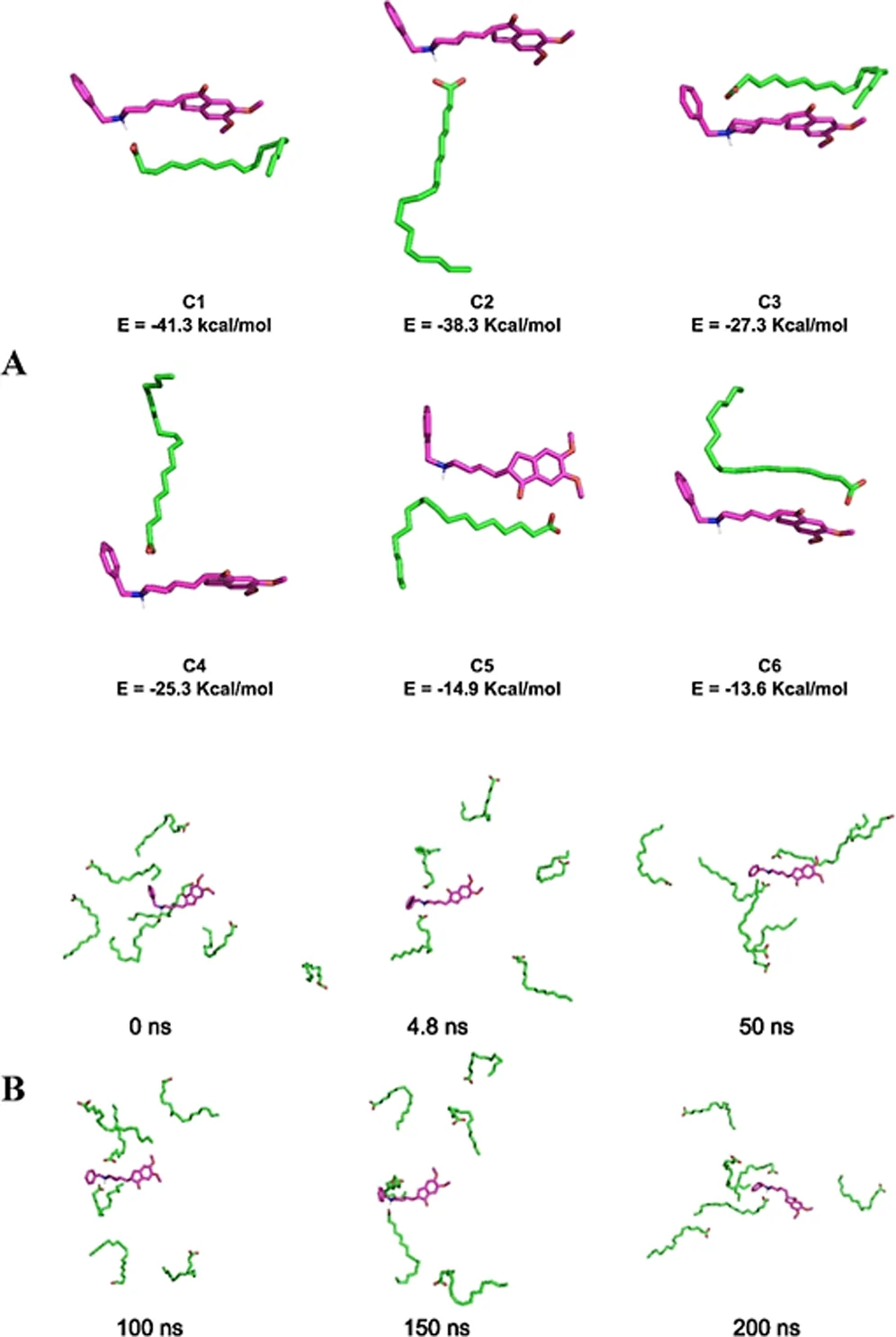
Molecular modeling of the donepezil–oleic acid (DP–OA) complex. (A) DOCKER analysis identified six distinct conformations of the DP–OA ion-paired complex. (B) Representative frames from the molecular dynamics simulation at 0, 4.8, 50, 100, 150, and 200 ns, illustrating the stability and spatial organization of the ion-paired complex over time.

The next two best scored complexes were **C3** (*E* = −27.3 kcal/mol) and **C4** (*E* = −25.3 kcal/mol) (Figure 2A). In these two complexes, OA is positioned on the other side of the donepezil ring system. For both, the distance of the oxygen atoms of OA from the hydrogens of the piperidine ring in donepezil is ∼2 Å, which could characterize a weak C–H···O hydrogen bonding (Figure 2B). It is likely that long-range electrostatic interactions are also contributing to the stability of **C3** and **C4**. Finally, **C5** and **C6** were the least stable complexes with interaction energies of −14.9 and −13.6 kcal/mol, respectively.

Since OA is a very flexible molecule whose conformation might be strongly influenced by its environment, we wondered which conformation of the OA-DP complex would be stable in solution. To answer this question, we conducted a molecular dynamics simulation for a system composed of one donepezil molecule and six OA molecules randomly distributed around the drug. The simulation was performed using chloroform as the solvent to simulate our NMR experiment (Supporting Information, Figure S12).

The simulation analysis showed that the OA molecules display, as expected, a high flexibility. Four of them never interacted with donepezil, but diffuse freely throughout the whole simulation time. On the other hand, two molecules of OA showed stable interactions with donepezil. The first one formed a hydrogen bond between the carboxylate of OA and the charged nitrogen of the piperidine ring of donepezil, which is present during the whole simulation (Figure 2B, and Supplementary Movie 1). At approximately 4.8 ns into the simulation, a second OA molecule approaches donepezil and maintains stable interactions with it for the remainder of the simulation. In this case, the carboxylate of OA interacts with the hydrogens attached to the carbons adjacent to the nitrogen atom of donepezil.

The complex observed during the MD simulations is similar to poses **C2** and **C4** obtained with DOCKER. Overall, and in agreement with our NMR results, these combined computational observations provide a direct indication of the formation of the OA-DP complex.

### 3.2 Formulations

Myverol 18–92 K and OA were selected because, upon hydration, they self-assemble into H_II_ mesophases suitable for nanodispersion formation. OA modulates lipid packing, favors the formation of H_II_ mesophases, and simultaneously acts as the counterion for hydrophobic ion-pair formation with donepezil. Poloxamer 407 was incorporated as a nonionic steric stabilizer to stabilize the nanoparticles generated during sonication by preventing aggregation and coalescence, thereby improving the colloidal stability of the liquid crystalline nanodispersion without disrupting its internal mesophase structure.^20,21^

The precursor liquid crystals loaded with donepezil, when observed by polarized light microscopy, indicated the formation of a hexagonal phase (Figure 3A,B). The hexagonal phase is a liquid-crystalline structure with unique optical properties. Its characteristic image pattern was identifiable due to its distinctive texture, exhibiting anisotropy, which indicates that its optical properties vary depending on the direction. Striated or folded structures were observed. This mesophase is birefringent, meaning it refracts light differently depending on the polarization direction, rotating the plane of polarized light and producing a colored image.^19,54,55^

**Figure 3:**
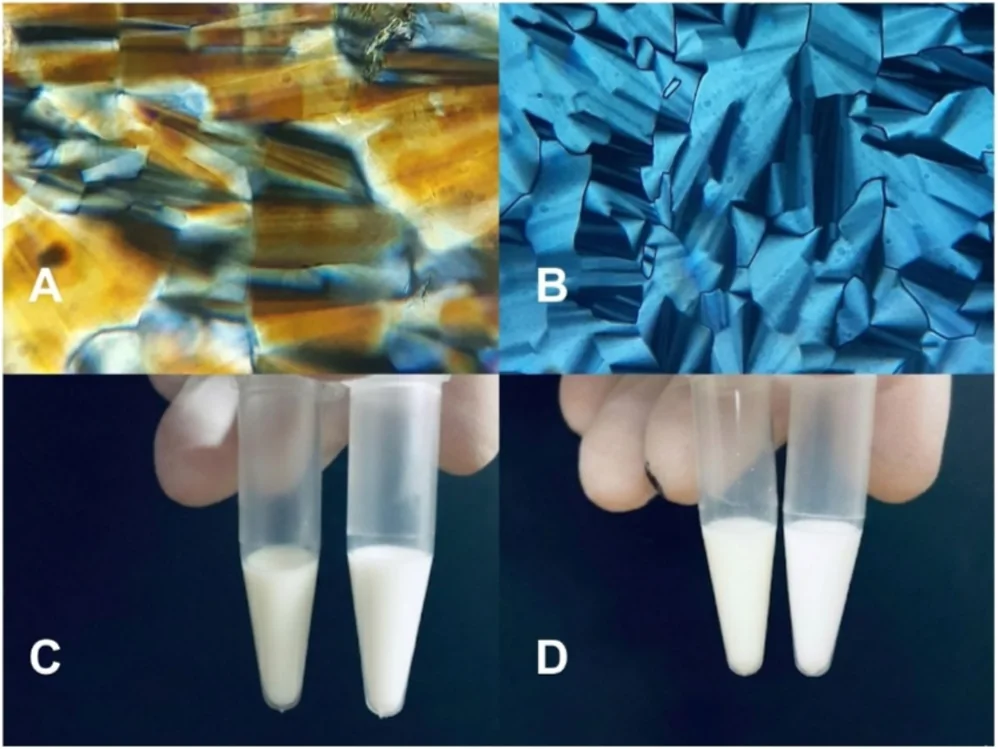
Polarized light microscopy (40×) images of donepezil-loaded liquid crystalline nanodispersions: (A) LCN-1 and (B) LCN-2. (C,D) Macroscopic appearance of the corresponding formulations with (LCN) and without donepezil (PLCN): (C) LCN-1/PLCN-1 and (D) LCN-2/PLCN-2.

After sonication, all formulations (LCNs containing donepezil and PLCNs without drug) exhibited similar macroscopic characteristics, including high fluidity and a white, opaque, homogeneous appearance, with no visible aggregates or signs of precipitation. No phase separation was observed after centrifugation, indicating physical stability consistent with colloidal dispersions. Moreover, no visual differences were detected between formulations with and without donepezil (Figure 3C,D).

The HPLC results for donepezil quantification indicated that no interfering peaks from the skin or the formulation matrix coeluted with donepezil at its retention time. Therefore, the analytical method proved to be selective and suitable for application at the various stages of this study, including drug encapsulation in LCNs, in vitro release assays, and skin absorption experiments (Supporting Information, Figure S14 and Table S3).

The donepezil content in LCN-1 and LCN-2 was 95.5 ± 0.9% and 107.8 ± 0.9%, respectively. Both formulations exhibited high EE, with values of 91.3 ± 0.8% for LCN-1 and 97.9 ± 0.2% for LCN-2, indicating that the majority of the drug was incorporated into the nanoparticles, with less than 10% remaining in the AP. The higher EE observed for LCN-2 was attributed to the presence of OA in the nanoparticles, which promotes ionic pairing with donepezil and thereby enhances drug encapsulation.

DL, expressed as % w/w, was also substantial, with values of 14.8 ± 0.1% for LCN-1 and 14.2 ± 0.2% for LCN-2. High DL is an important parameter for achieving clinically relevant doses while minimizing the volume of formulation required.

Donepezil-containing formulations exhibited higher pH values (8.02 and 7.02 for LCN-1 and LCN-2, respectively) compared to drug-free formulations (6.78 and 6.27 for PLCN-1 and PLCN-2, respectively). The reduction in pH observed for OA-containing systems is consistent with the presence of a fatty acid in the formulation. Conversely, the increase in pH in drug-loaded systems can be attributed to the basic nature of donepezil, which contains a tertiary amine (*pK*_a_ ≈ 8.9) and can partially accept protons, thereby influencing the pH of the aqueous phase. Under these conditions, donepezil is expected to be predominantly in its protonated form, which favors electrostatic interactions with OA, supporting the formation of ion pairs within the lipid matrix.

The LCNs were formulated with pH values close to neutrality to favor donepezil encapsulation and chemical stability, while also considering adequate skin tolerability.^56,57^ Although the natural pH of the skin is slightly acidic (∼5.5), which is considered optimal for topical application, the low electrolyte concentration in the AP (5 mM) allows for the potential future application of these formulations to mucosal tissues.

Rheological analysis indicated Newtonian behavior for all formulations, with apparent viscosities ranging from 2.9 to 4.0 mPa s and no significant dependence on shear rate (Figure 4, *p* > 0.05). Although the developed formulations exhibited Newtonian flow behavior, this characteristic does not necessarily limit their applicability for transdermal delivery. It is important to emphasize that the present systems should be regarded as prototype nanodispersions, intended to be further incorporated into suitable transdermal platforms, such as adhesive patches or gel-based matrices.^7^ In such systems, the residence time and skin contact are governed primarily by the structural and adhesive properties of the final dosage form rather than by the intrinsic viscosity of the nanodispersion itself. Therefore, the Newtonian behavior observed here is not expected to compromise drug retention under practical conditions, as prolonged skin contact would be ensured by the final formulation design.

**Figure 4:**
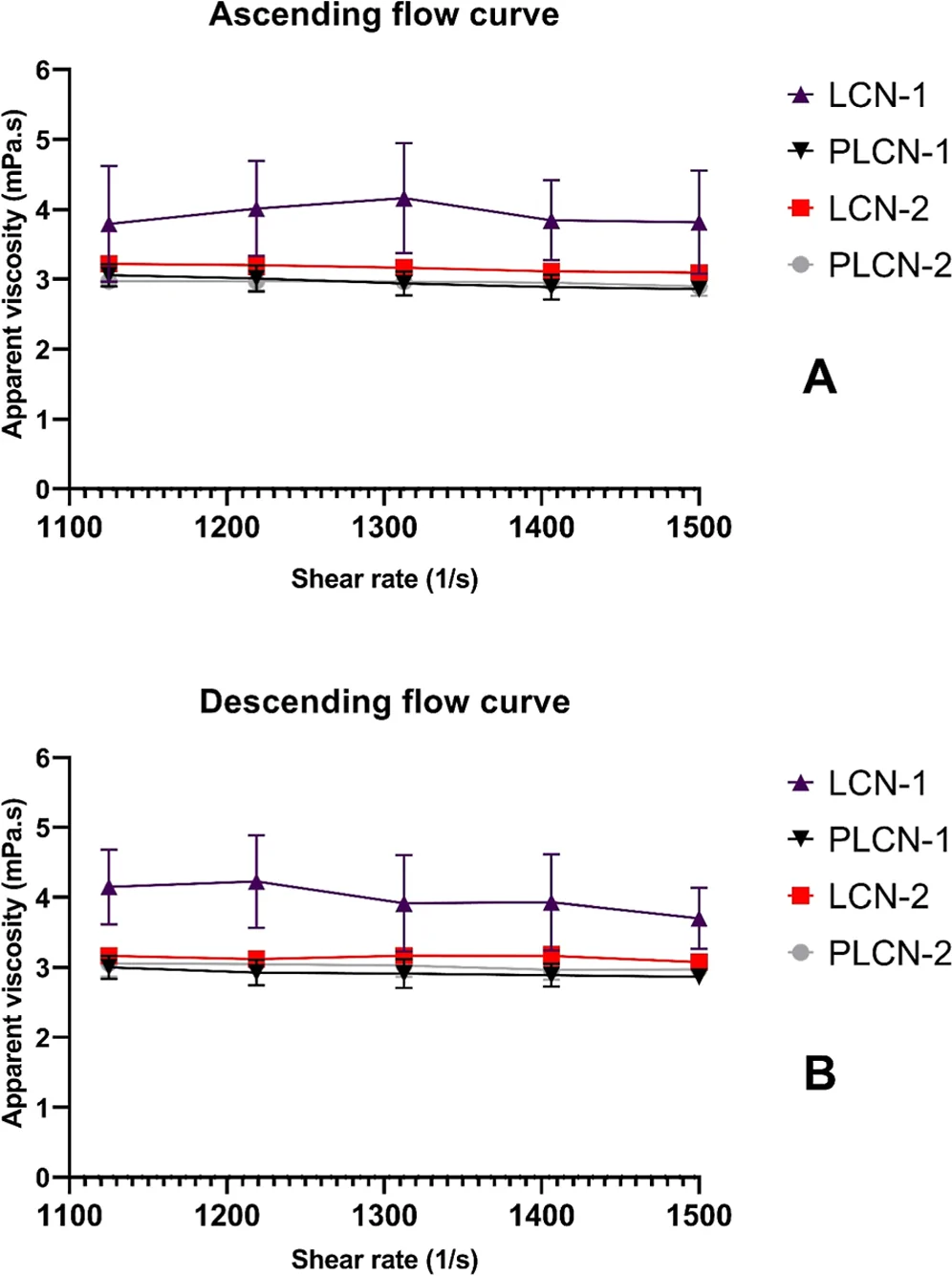
Rheological behavior of formulations with (LCN) and without donepezil (PLCN), LCN-1, PLCN-1, LCN-2, and PLCN-2 (*n* = 3): (A) ascending (up-sweep) flow curves and (B) descending (down-sweep) flow curves.

Among the groups, drug-free formulations, PLCN-1 and PLCN-2, showed apparent viscosities between 2.9 and 3.0 mPa s, with no significant differences in either the ascending or descending flow curves. LCN-2 (with donepezil) did not differ from PLCN-2 in the descending curve; however, a significant difference in apparent viscosity was observed in the ascending curve. LCN-1 (with donepezil) exhibited the highest viscosities, close to 3.9–4.0 mPa s, significantly differing from the other groups (*p* < 0.05). All other comparisons showed no significant differences, with LCN-2, PLCN-2, and PLCN-1 maintaining apparent viscosities between 2.9 and 3.0 mPa s. The high fluidity of these formulations allows for subsequent viscosity modulation through the incorporation of gelling agents, providing flexibility for further formulation development.^58^

After sonication, the HD differed markedly among the formulations. The drug-free formulation without OA (PLCN-1) and drug-loaded formulation without OA (LCN-1, with donepezil) presented HD values of 328.7 ± 3.1 nm and 242.8 ± 4.7 nm, respectively, whereas smaller particle sizes were obtained for drug-free formulation with OA (PLCN-2) (198.7 ± 0.8 nm) and drug-loaded formulation with OA (LCN-2) (183.0 ± 1.5 nm). A similar trend was observed for the PDI, with higher values for PLCN-1 (0.464 ± 0.024) and LCN-1 (0.361 ± 0.036) compared with PLCN-2 (0.225 ± 0.001) and LCN-2 (0.183 ± 0.006). These results indicate a narrower size distribution for the OA-containing systems (PLCN-2 and LCN-2). The presence of OA can interfere with the internal liquid-crystalline organization of the nanostructures by altering lipid packing parameters related to the H_II_ phase. Such changes in molecular packing and interfacial curvature are closely associated with particle size and may facilitate the formation of smaller nanodispersions, consistent with the reduced HD observed for LCN-2 and PLCN-2 ^21^.

The ZP reflected differences in lipid composition. The drug-free formulation without OA (PLCN-1) and drug-loaded formulation without OA (LCN-1, with donepezil) exhibited moderately negative ZP values (−12.4 ± 0.3 mV and −19.9 ± 0.3 mV, respectively), whereas substantially more negative values were observed for drug-free formulation with OA (PLCN-2) (−29.8 ± 0.5 mV) and drug-loaded formulation with OA (LCN-2) (−30.7 ± 0.3 mV). Based on these results, donepezil incorporation had minimal influence on the surface charge. The higher negative ZP of LCN-2 and PLCN-2 is attributed to the presence of OA, which is negatively charged and significantly contributes to the electrokinetic properties of the nanodispersions, potentially enhancing colloidal stability through increased electrostatic repulsion. Monoglycerides, the main lipid component of Myverol 18–92 K used in all formulations, are electrically neutral and contributes minimally to electrostatic interactions, although they govern the formation of the liquid crystalline matrix. Thus, variations in particle size are more closely related to changes in lipid packing and mesophase organization, whereas differences in ZP primarily reflect changes in surface charge density.

The variation in HD, PDI, and ZP over a 90 day period, with measurements every 15 days, was a key criterion for selecting formulations with improved physical stability. Regarding HD (Figure 5AB), greater variability was observed for LCN-1 stored under refrigerated conditions, likely due to possible lipid crystallization at low temperatures.^59^ In contrast, LCN-2, PLCN-1, and PLCN-2 showed only minor fluctuations in HD over time.

**Figure 5:**
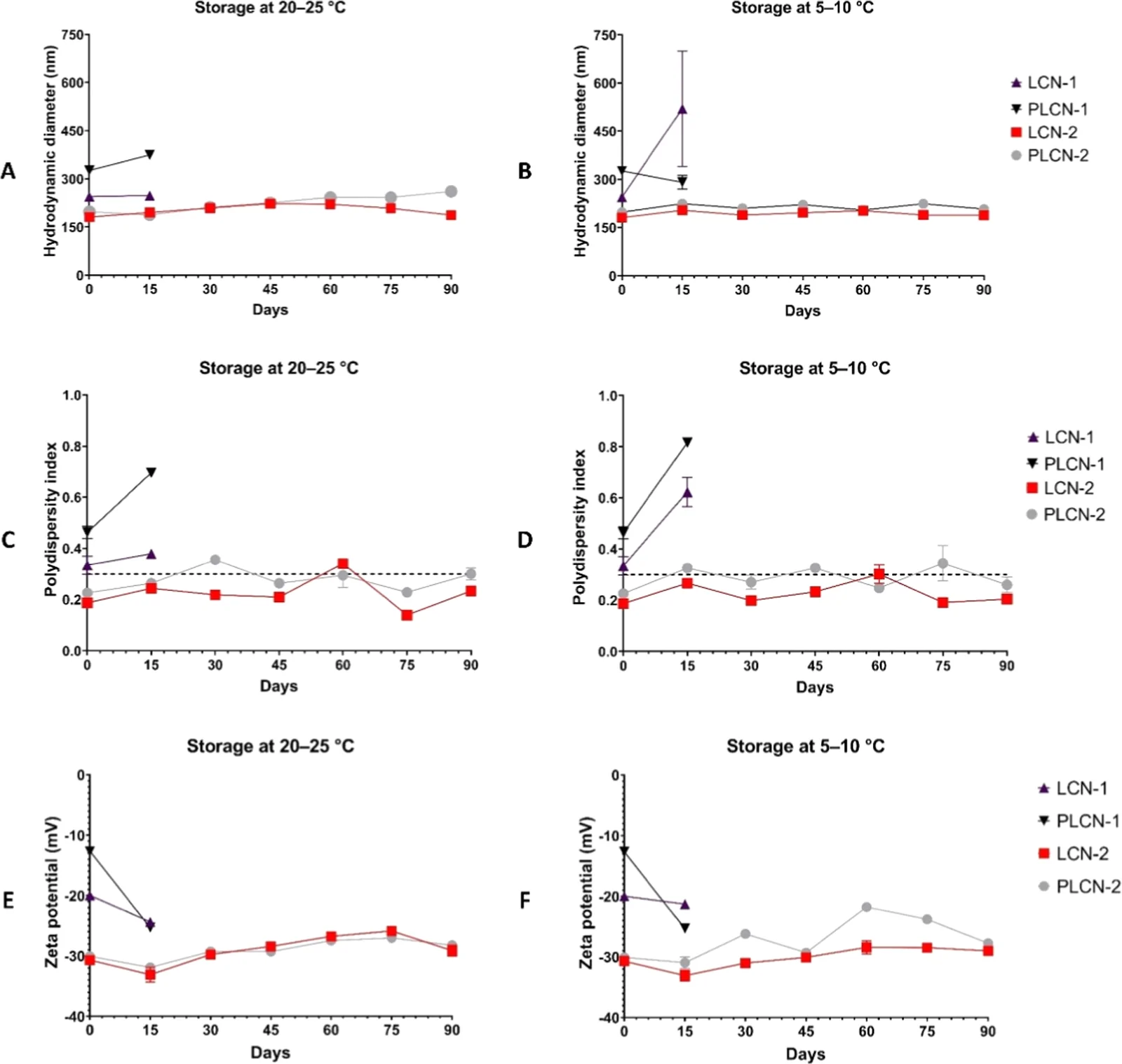
Physical stability of formulations with (LCN) and without donepezil (PLCN), LCN-1, PLCN-1, LCN-2, and PLCN-2 during storage at 20–25 °C and 5–10 °C (*n* = 3): (A,B) hydrodynamic diameter; (C,D) polydispersity index; (E,F) zeta potential.

Regarding the PDI (Figure 5C and D), LCN-2 exhibited lower variability compared with the other formulations. PLCN-1 and LCN-1 exhibited a tendency for PDI values to exceed 0.3 within 15 days, indicative of physical instability; consequently, stability monitoring of these formulations was discontinued. In contrast, LCN-2 maintained PDI values below 0.3 during storage at room temperature, with only a transient increase observed at 60 days.

Rapid crystallization of unsaturated monoglycerides in colloidal dispersions upon cooling to 4 °C has been previously reported.^59,60^ OA is a liquid lipid, and its incorporation into LCNs increases lipid matrix fluidity, thereby reducing the tendency toward crystallization upon temperature reduction. Overall, LCN-2 demonstrated superior physical stability compared to LCN-1, which can be attributed to the presence of OA in LCN-2 and its absence in LCN-1.

ZP measurements performed for the formulations revealed similar overall trends (Figure 5EF). PLCN-1 and LCN-1 exhibited a gradual reduction in ZP within the first 15 days, regardless of storage temperature. In contrast, PLCN-2 and LCN-2 showed only minor ZP variations during storage at room temperature over 90 days, while more pronounced changes were observed for PLCN-2 under refrigerated conditions. Notably, LCN-2 displayed higher ZP values than LCN-1, likely due to the contribution of the negatively charged OA. This increased surface charge may enhance electrostatic repulsion between particles, contributing to improved colloidal stability and the lower PDI values observed for LCN-2.

### 3.3 In Vitro Release and Skin Absorption Studies

Sodium acetate buffer (pH 4.5) was used as the receptor medium in the in vitro release and skin absorption assays of donepezil, as this pH is compatible with cutaneous administration. The receptor compartment was filled with acetate buffer (pH 4.5), which ensured appropriate sink conditions throughout the experiment.^5,19^ The physiological pH of the skin surface is mildly acidic, typically ranging from approximately 4.5 to 5.5, with an average value close to 4.7.^61,62^ This acidic environment, commonly referred to as the acid mantle, plays a crucial role in maintaining stratum corneum integrity, lipid organization, antimicrobial defense, and barrier function. Therefore, formulations with pH values close to the physiological skin pH are generally considered advantageous for topical and transdermal administration.

The pH adjustment of the receptor medium is a critical factor, as donepezil exhibits pH-dependent solubility, with increased solubility under acidic conditions. Under these conditions, donepezil remains in its free form, enabling reproducible diffusion behavior. As previously demonstrated by our group, these conditions are suitable for evaluating donepezil release using vertical diffusion cells.^5^

The control solution exhibited a drug content of 96.0 ± 0.1%, and donepezil readily diffused across the dialysis membrane during the in vitro release studies (Figure 6A). This behavior reflects the complete solubilization of donepezil in the donor vehicle, indicating that drug diffusion was not significantly limited by the dialysis membrane under the experimental conditions. Collectively, these findings confirm that the selected receptor medium and release conditions were suitable for evaluating the release performance of the investigated formulations.

**Figure 6:**
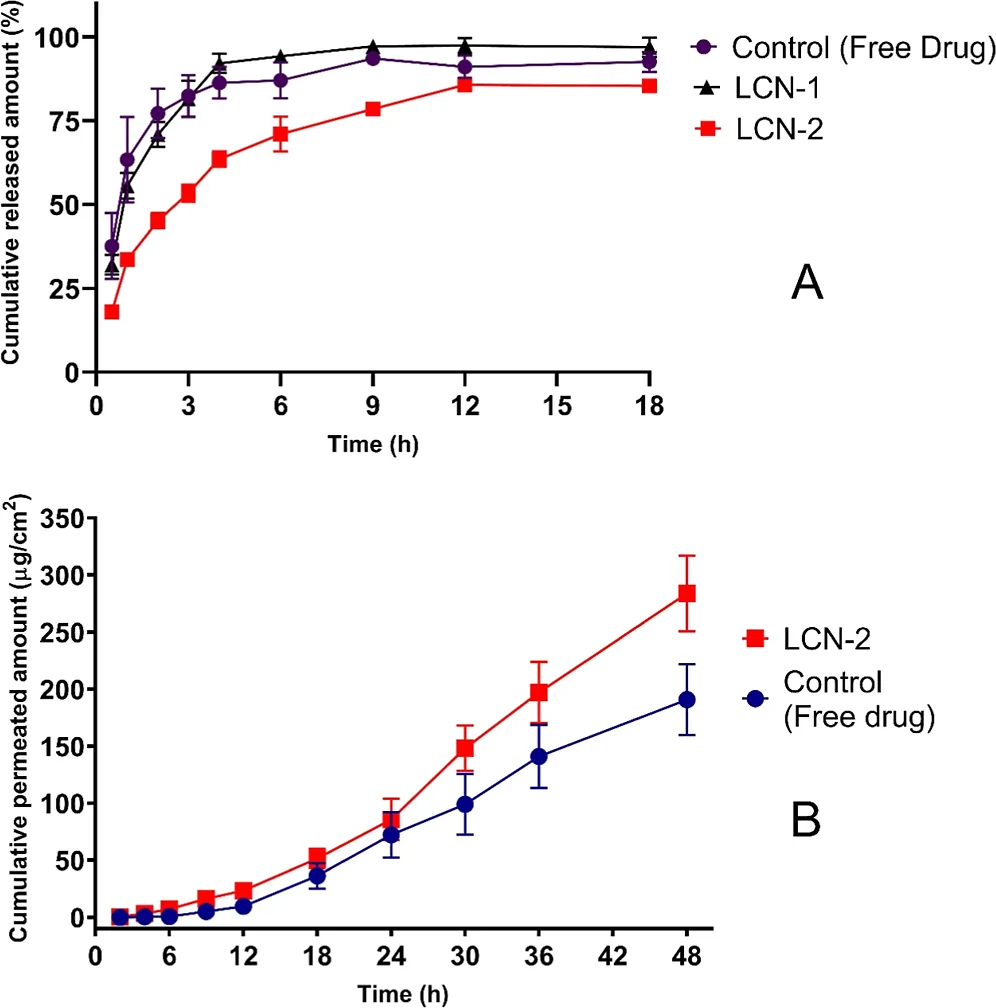
Donepezil profiles (*n* = 6): (A) in vitro release in sodium acetate buffer (pH 4.5) at 37 °C from the control, LCN-1 and LCN-2; (B) skin permeation from the control and LCN-2. The receptor chamber was filled with sodium acetate buffer (pH 4.5) and maintained at 37 °C.

Accordingly, the control formulation exhibited rapid drug release, with 63.4 ± 12.7% of donepezil released within 1 h and 86.4 ± 4.7% within 4 h (Figure 6A), as expected for a fully solubilized drug. These results also indicate that the dialysis membrane imposed minimal diffusional resistance, supporting its suitability for evaluating formulation performance.

LCN-1 showed a similarly rapid release profile, reaching 55.6 ± 3.9% within 1 h and 92.2 ± 2.9% within 4 h, with a plateau observed after approximately 4 h. Notably, the overall shape of the release curves for LCN-1 and the control was highly similar, with both systems exhibiting a steep initial release followed by a plateau phase. Both formulations achieved more than 85–90% drug release within the first 4 h, indicating that LCN-1 did not provide a significant barrier to drug diffusion.

Given this comparable release behavior, no meaningful differences in release kinetics could be established between LCN-1 and the control. Consequently, the application of mathematical models was not appropriate for LCN-1, as the assumptions of controlled or diffusion-limited release were not met. These findings confirm that LCN-1 does not provide controlled drug release and highlight the critical role of OA in modulating release behavior, as observed for LCN-2.

LCN-2 exhibited a modified release profile of donepezil (Figure 6A). Drug release was slower and more gradual, reaching more than 85% after 12 h, followed by the establishment of a plateau. This behavior indicates that LCN-2 provides a modulated release profile compared with the control formulation. Such modulation may be advantageous for transdermal delivery, as it can reduce initial drug burst and limit skin exposure to high drug concentrations.

The ∼1:2 molar ratio of donepezil to OA favors ion-pair formation, as supported by NMR data (NOESY/ROESY and DOSY), indicating close intermolecular interactions and reduced drug mobility within the lipid matrix. This restricted mobility provides a mechanistic basis for the modulation of drug release observed for LCN-2. Consistent with this, the Korsmeyer–Peppas model applied to LCN-2 (up to 12 h, <60% release) showed good fitting (*r* = 0.9961 ± 0.0014) and yielded an *n* value of 0.79 ± 0.03. This value, characteristic of anomalous (non-Fickian) transport, reflects a shift from simple diffusion toward a combined mechanism involving diffusion and matrix relaxation. Notably, this shift in *n* can be directly associated with the intermolecular interactions between donepezil and OA, which constrain drug diffusion and promote a more complex release mechanism.

Controlled drug release is critical for enhancing the safety and reproducibility of transdermal formulations, particularly for systems operating near saturation, as it prevents excessive drug exposure at the skin surface.^9^ Similar behavior has been reported for donepezil-loaded lipid nanocarriers prepared with fatty acids, including OA and stearic acid.^58^ In that system, drug release followed a non-Fickian mechanism (*n* ≈ 0.83), as determined by the Korsmeyer–Peppas model, which can be attributed to intermolecular interactions between donepezil and ionizable lipid components, analogous to those observed in the present study. In contrast, studies on progesterone-loaded LCNs have reported lower *n* values (0.461–0.586) under similar experimental conditions.^59^ This difference can be rationalized by the physicochemical nature of progesterone, a neutral molecule with limited ability to establish strong intermolecular interactions with the lipid matrix (e.g., glyceryl monooleate). As a result, drug diffusion is less constrained, leading to a release mechanism closer to Fickian transport. Taken together, these findings reinforce that the higher *n* value observed in the present study arises from specific interactions between donepezil and OA, which restrict drug mobility within the nanostructure and promote a more complex, non-Fickian release behavior.

Skin absorption studies provide information on both drug permeation across the skin and skin retention, defined as the fraction of the applied dose retained within the tissue. Porcine ear skin is widely accepted as an ex vivo surrogate for human skin because of its close biochemical, histological, biophysical, and permeability characteristics, including a comparable stratum corneum barrier and similar permeation behavior, particularly for lipophilic compounds. Moreover, it is readily available, highly reproducible, and does not require ethical approval, making it a reliable model for evaluating transdermal drug delivery systems.^57,63–65^ LCN-2, which demonstrated the ability to modulate donepezil release kinetics, was selected for comparison with a control solution consisting of donepezil dissolved in acetate buffer (pH 4.5) containing 6.7% ethanol (Figure 6B). This control formulation enables the solubilization of donepezil at the same concentration used in the LCNs.

The permeation flux of LCN-2 was 7.5 ± 1.1 μg cm^–2^ h^–1^, as determined by fitting the data to a zero-order kinetic model over the 12–48 h interval, yielding an excellent correlation coefficient (*r* = 0.9908 ± 0.0074) (Figure 6B). The *T*_lag_, defined as the period between formulation application and the onset of steady-state permeation, was 10.32 ± 1.67 h. In comparison, the control solution exhibited a permeation flux of 5.2 ± 0.8 μg·cm^–2^·h^–1^, also adequately described by a zero-order model (r = 0.9921 ± 0.0051), with a *T*_lag_ of 10.31 ± 1.62 h. A statistically significant increase in permeation flux was observed for LCN-2 compared with the control (*p* = 0.0011), whereas no significant difference was found in *T*_lag_ (*p* = 0.4959). The calculated *K*_p_ × 10^–5^ values were 94.0 ± 14.3 cm/h and 64.6 ± 10.0 cm/h for LCN-2 and control, respectively. After 48 h, 24% of the applied dose was absorbed from LCN-2, compared with 16% from the control formulation. These results indicate that LCN-2 enhances the extent and rate of donepezil permeation without altering the onset of steady-state transport.

The increased permeation of donepezil is likely attributable to the presence of OA in LCN-2, which acts as a chemical skin permeation enhancer by increasing stratum corneum permeability. In addition, the structural features of the LCNs, including their hydrophilic and hydrophobic domains, may further facilitate drug penetration. Particle charge primarily contributes to colloidal stability, while the permeation-enhancing effects of OA and monoolein are considered key drivers of transdermal drug delivery.^66^ It is important to note that the control solution contains a small amount of ethanol (6.7%), which may act as a permeation enhancer. Nevertheless, the permeation flux of LCN-2 was approximately 45% higher than that of the control solution.

The theoretical steady-state plasma concentration of donepezil following transdermal administration was estimated using a simplified pharmacokinetic approach described for transdermal drug delivery.^67,68^ First, the systemic input rate (*R*_in_) was calculated from the experimentally determined steady-state transdermal flux (*J*_ss_) according to eq 10

10 $$R_{in}=J_{ss}\times A$$

where *J*_ss_ is the steady-state transdermal flux (μg cm^–2^ h^–1^) and (*A*) is the effective application area (cm^2^). Application areas of 10, 20, and 30 cm^2^ were considered for the simulations. Simulations were performed assuming a 70 kg adult.

Assuming complete systemic absorption and linear pharmacokinetics, the theoretical steady-state plasma concentration (*C*_ss_) was estimated according to eq 11

11 $$C_{ss}=R_{in}/CL$$

where (CL) is the systemic clearance.

The measured transdermal flux of 7.5 ± 1.1 μg cm^–2^ h^–1^ would theoretically provide steady-state plasma concentrations of approximately 8.2, 16.5, and 24.7 ng/mL for transdermal systems with effective application areas of 10, 20, and 30 cm^2^, respectively. Since intravenous pharmacokinetic clearance data for donepezil were not available, the apparent oral clearance (CL/F) reported in the literature (0.13 L h^–1^ kg^–1^) was used for these simulations.^69^ This approach is justified because donepezil exhibits rapid absorption and an oral bioavailability approaching 100%, making the reported CL/F a reasonable approximation of systemic clearance.

The predicted steady-state plasma concentrations are within a clinically relevant range and are comparable to plasma concentrations reported following oral administration of donepezil. For example, single oral doses of 2, 4, and 6 mg produce mean peak plasma concentrations (*C*_max_) of 3.2, 6.9, and 11.6 ng/mL, respectively.^69^ Notably, the simulated steady-state concentration for a 10 cm^2^ transdermal system (8.2 ng/mL) is comparable to the Cmax achieved after a 6 mg oral dose, whereas larger application areas are predicted to produce proportionally higher steady-state concentrations.

It should be emphasized, however, that these parameters are not directly equivalent. Oral administration results in fluctuating plasma concentrations characterized by repeated peak (*C*_max_) and trough (C min) levels following each dose, whereas transdermal delivery is expected to provide a nearly constant rate of drug input, maintaining a relatively stable steady-state plasma concentration. Consequently, transdermal administration may achieve therapeutic drug exposure while minimizing peak-to-trough fluctuations, potentially reducing concentration-dependent adverse effects and improving long-term treatment adherence.

Skin retention of donepezil was quantified at 24 and 48 h, and no statistically significant differences were observed between LCN-2 and the control formulation (*p* = 0.5471). In both cases, donepezil levels in the skin remained relatively constant over time, indicating that steady-state conditions were achieved at 24 h and maintained up to 48 h. The control formulation exhibited retention values of 8.8 ± 8.3% and 6.9 ± 6.9% at 24 and 48 h, respectively, whereas LCN-2 showed slightly lower values of 6.0 ± 1.9% and 4.2 ± 1.3% at the same time points. Despite the absence of significant differences in the extent of skin retention, the control formulation showed markedly higher variability, which can be attributed to its rapid and unmodulated drug release profile. In contrast, the more consistent retention observed for LCN-2 suggests that modulation of drug release contributes to a more controlled and reproducible interaction with the skin, even without increasing the total amount of drug retained.

Clinical studies have shown that transdermal donepezil systems (Corplex, 5–10 mg/day) achieve bioequivalence to daily oral dosing while maintaining a favorable safety profile.^11^ These results underscore the potential of advanced transdermal technologies, such as LCN-2, to improve both the efficacy and safety of cutaneous donepezil therapy.

### 3.4 Preliminary Cytotoxicity

The preliminary cytotoxicity of donepezil and the developed formulation (LCN-2) was evaluated according to ISO 10993-5:2009, which defines a substance as cytotoxic when cell viability falls below 70% (dashed line in Figure 7). Cell viability in MRC-5 fibroblasts was assessed after 24 h of exposure to various concentrations of donepezil and LCN-2. For LCN-2, the tested concentrations corresponded to approximately 125 times the equivalent donepezil content, considering a drug concentration of 0.8% w/v.

**Figure 7:**
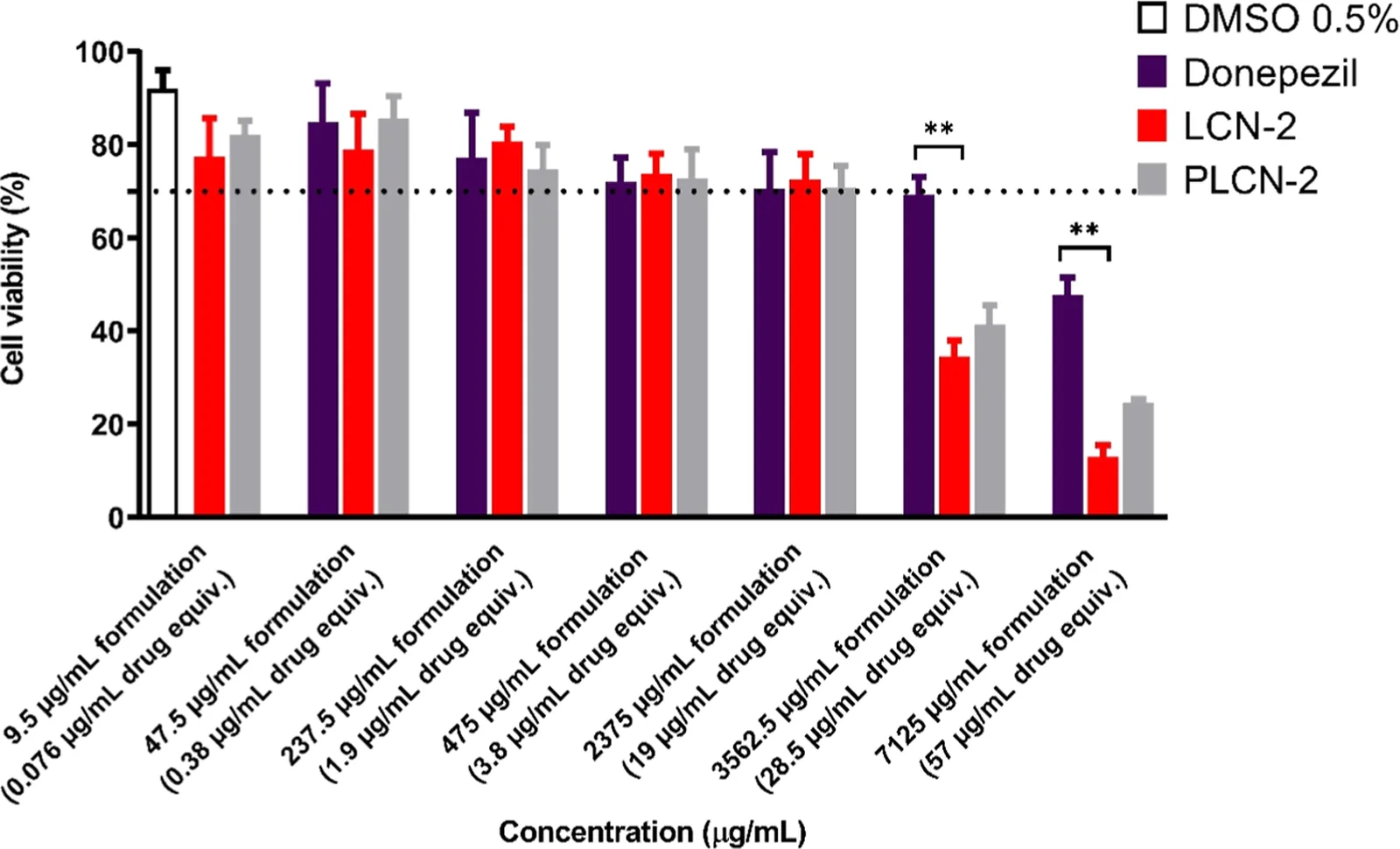
Preliminary cytotoxicity in MRC-5 cells (*n* = 5). Donepezil was tested at concentrations of 0.38, 1.9, 3.8, 19.0, 28.5, and 57 μg mL^–1^. LCN-2 and placebo (PLCN-2) were tested at formulation concentrations equivalent to these amounts of drug.

Although lung fibroblasts (MRC-5 cell line) are not representative of skin tissue, they provide a robust and widely accepted in vitro model for preliminary cytotoxicity screening. In this study, their use was intended to assess the basal biocompatibility and potential systemic toxicity of the developed nanodispersions, rather than to mimic the skin barrier. Although derived from lung tissue, these human diploid fibroblasts are commonly employed as a general surrogate for stromal cell biocompatibility and provide valuable insight into basal cellular responses to nanocarriers. In this context, fibroblasts are key extracellular matrix–producing cells, and MRC-5 cultures have been shown to synthesize and interact with matrix proteins such as fibronectin, thereby enabling assessment of potential perturbations in cell–matrix homeostasis in vitro.^70,71^ Therefore, these findings should be interpreted as an initial safety assessment, and further studies using skin-relevant models (e.g., keratinocytes or reconstructed skin equivalents) are required to confirm dermal compatibility.

At lower concentrations, cell viability remained above 70% for donepezil, LCN-2, and its placebo, indicating biocompatibility. Notably, LCN-2 maintained >70% viability at concentrations up to 2375.0 μg/mL, equivalent to 19 μg/mL of donepezil, suggesting biocompatibility even at one of the higher tested doses. As concentrations increased further, viability progressively decreased, falling below 70% at 28.5 μg/mL of donepezil and 3562.5 μg/mL of LCN-2, and dropping below 40% at the highest tested concentrations (57 μg/mL donepezil and 7125 μg/mL LCN-2). The safety profile of the LCN-2 is in agreement with previous reports describing the biocompatibility of nanoparticle-based delivery systems evaluated in MRC-5 cells.^70,72,73^ Notably, several nanoscale platforms have demonstrated limited cytotoxic effects at relevant exposure concentrations, supporting the use of this cell line as a sensitive model for early stage screening of nanostructured carriers.

The cytotoxicity of donepezil and OA–conjugated PAMAM G4 dendrimers loaded with the drug was evaluated in SH-SY5Y cells, demonstrating good safety and cytocompatibility over a broad concentration range.^74^ Cell viability remained above 70% at concentrations up to 25 μg/mL of free donepezil, but declined markedly at concentrations of 50 μg/mL and above, in agreement with the findings observed in the present study.

Cytotoxicity assays using MRC-5 fibroblasts showed a concentration-dependent reduction in cell viability. While viability remained above 70% for most tested concentrations, a decrease below this threshold was observed at the highest LCN-2 levels (>2000.0 μg/mL). This effect likely associated with the high nanoparticle density rather than with intrinsic toxicity of donepezil itself. However, it is important to note that these concentrations (up to 2375.0 μg/mL) significantly exceed the expected therapeutic range for transdermal exposure. At lower, clinically relevant doses, LCN-2 demonstrated a more favorable safety profile. Therefore, while high concentrations of these OA-based LCNs can induce modest cytotoxicity, our findings suggest preliminary biocompatibility within the intended practical exposure range, supporting further investigation for transdermal delivery.

## 4 Conclusion

This study demonstrates that hydrophobic ion-pair formation between donepezil and OA is an effective strategy for modulating the performance of liquid crystalline nanodispersions intended for transdermal delivery. The combined NMR, Raman spectroscopy, molecular docking, and molecular dynamics findings consistently supported the formation of hydrophobic ion pairs between donepezil and OA. Incorporation of OA improved encapsulation efficiency, reduced particle size and polydispersity, enhanced colloidal stability, and promoted a more sustained drug release profile. These physicochemical improvements translated into enhanced skin permeation, resulting in an approximately 45% higher steady-state flux than that achieved with the control donepezil solution, while maintaining acceptable in vitro cytocompatibility. Overall, hydrophobic ion-pair formation represents a simple and effective formulation strategy to optimize lipid-based nanocarriers for the transdermal delivery of donepezil and may also be applicable to other weakly basic drugs.

## Supplementary Material





## Notes

Movie_01.mp4 (MP4)
Supplementary material with figures—R3 (PDF)

## References

[1] Alzheimer’s
Association (2021). 2021 Alzheimer’s
Disease Facts and Figures. Alzheimer’s
Dement.

[2] Zhao Z. Q., Chen B. Z., Zhang X. P., Zheng H., Guo X. D. (2021). An Update
on the Routes for the Delivery of Donepezil. Mol. Pharmaceutics.

[3] Moffat, A. C. ; Osselton, M. D. ; Widdop, B. Drugs and Poisons. Clarke’s Anal. drug poisons Pharm. body fluids postmortem Mater, 4th ed.; Pharmaceutical Press: London, 2009; p 2609.

[4] Asiri, Y. A. ; Mostafa, G. A. E. Donepezil. In Profiles of Drug Substances, Excipients and Related Methodology; Brittain, H. G. , Ed.; Academic Press Inc., 2010; Vol. 35, pp 117–150. 10.1016/S1871-5125(10)35003-5 22469221

[5] Gomides
T G. F., Marques V C., Silva P P., Fernandes G R. R., Jammal N F., Marques F S., Barboza A P. M., Vieira P M. A., de Souza J., de Souza Filho J. D., Ruela L. M. (2025). A. The Role of Intermolecular Interactions between
Donepezil and Methacrylic Acid in the Synthesis of Crosslinked Polymer
Microgels for Drug Delivery. J. Braz. Chem.
Soc..

[6] Choi J., Choi M. K., Chong S., Chung S. J., Shim C. K., Kim D. D. (2012). Effect of Fatty Acids on the Transdermal
Delivery of
Donepezil: In Vitro and in Vivo Evaluation. Int. J. Pharm..

[7] Mendes I. T., Ruela A. L. M., Carvalho F. C., Freitas J. T. J., Bonfilio R., Pereira G. R. (2019). Development and Characterization
of Nanostructured
Lipid Carrier-Based Gels for the Transdermal Delivery of Donepezil. Colloids Surf., B.

[8] Kaur P., Kaur A., Rimpi (2026). Advancements in Transdermal Drug
Delivery Using Ionic
Liquids: Design Strategies, Functional Roles, Mechanisms, and Future
Challenges. Intell. Pharm..

[9] Watkinson A. C. (2013). A Commentary
on Transdermal Drug Delivery Systems in Clinical Trials. J. Pharm. Sci..

[10] Di
Stefano A., Sozio, Cerasa, Marinelli (2012). Transdermal Donepezil on the Treatment of Alzheimer’s
Disease. Neuropsychiatr. Dis. Treat..

[11] Tariot P. N., Braeckman R., Oh C. (2022). Comparison of Steady-State Pharmacokinetics
of Donepezil Transdermal Delivery System with Oral Donepezil. J. Alzheimer’s Dis..

[12] Ristroph K., Salim M., Wilson B. K., Clulow A. J., Boyd B. J., Prud’homme R. K. (2021). Internal Liquid Crystal Structures in Nanocarriers
Containing Drug Hydrophobic Ion Pairs Dictate Drug Release. J. Colloid Interface Sci..

[13] Choi S. H., Park T. G. (2000). Hydrophobic Ion Pair Formation between
Leuprolide and
Sodium Oleate for Sustained Release from Biodegradable Polymeric Microspheres. Int. J. Pharm..

[14] Ristroph K. D., Prud’homme R.
K. (2019). Hydrophobic Ion Pairing:
Encapsulating Small Molecules,
Peptides, and Proteins into Nanocarriers. Nanoscale
Adv..

[15] Notman R., Anwar J. (2013). Breaching the Skin
Barrier - Insights from Molecular Simulation of
Model Membranes. Adv. Drug Delivery Rev..

[16] Hosmer J. M., Steiner A. A., Lopes L. B. (2013). Lamellar
Liquid Crystalline Phases
for Cutaneous Delivery of Paclitaxel: Impact of the Monoglyceride. Pharm. Res..

[17] Libster D., Aserin A., Garti N. (2011). Interactions of Biomacromolecules
with Reverse Hexagonal Liquid Crystals: Drug Delivery and Crystallization
Applications. J. Colloid Interface Sci..

[18] Borné J., Nylander T., Khan A. (2001). Phase Behavior
and Aggregate Formation
for the Aqueous Monoolein System Mixed with Sodium Oleate and Oleic
Acid. Langmuir.

[19] Ruela A. L. M., Carvalho F. C., Pereira G. R. (2016). Exploring the Phase Behavior of Monoolein/Oleic
Acid/Water Systems for Enhanced Donezepil Administration for Alzheimer
Disease Treatment. J. Pharm. Sci..

[20] Lopes L. B., Ferreira D. A., De Paula D., Garcia M. T. J., Thomazini J. A., Fantini M. C. A., Bentley M. V. L. B. (2006). Reverse
Hexagonal Phase Nanodispersion
of Monoolein and Oleic Acid for Topical Delivery of Peptides: In Vitro
and in Vivo Skin Penetration of Cyclosporin A. Pharm. Res..

[21] Nakano M., Teshigawara T., Sugita A., Leesajakul W., Taniguchi A., Kamo T., Matsuoka H., Handa T. (2002). Dispersions
of Liquid Crystalline Phases of the Monoolein/Oleic Acid/Pluronic
F127 System. Langmuir.

[22] Milak S., Zimmer A. (2015). Glycerol Monooleate Liquid Crystalline Phases Used
in Drug Delivery Systems. Int. J. Pharm..

[23] Wu H., Fang F., Zheng L., Ji W., Qi M., Hong M., Ren G. (2020). Ionic Liquid Form of
Donepezil: Preparation,
Characterization and Formulation Development. J. Mol. Liq..

[24] Zhao H., Lu, Gong T., Zhang Z. (2013). Nanoemulsion
Loaded with Lycobetaine–Oleic Acid Ionic Complex:
Physicochemical Characteristics, in Vitro, in Vivo Evaluation, and
Antitumor Activity. Int. J. Nanomedicine.

[25] Mittapelly N., Thalla M., Pandey G., Banala V. T., Sharma S., Arya A., Mishra S., Mitra K., Shukla S., Mishra P. R. (2017). Long Acting Ionically Paired Embonate Based Nanocrystals
of Donepezil for the Treatment of Alzheimer’s Disease: A Proof
of Concept Study. Pharm. Res..

[26] Kearney M.-C., Caffarel-Salvador E., Fallows S. J., McCarthy H. O., Donnelly R. F. (2016). Microneedle-Mediated
Delivery of Donepezil: Potential for Improved Treatment Options in
Alzheimer’s Disease. Eur. J. Pharm. Biopharm..

[27] Kim J.-Y., Han M.-R., Kim Y.-H., Shin S.-W., Nam S.-Y., Park J.-H. (2016). Tip-Loaded Dissolving
Microneedles for Transdermal
Delivery of Donepezil Hydrochloride for Treatment of Alzheimer’s
Disease. Eur. J. Pharm. Biopharm..

[28] Nayak A. S., Chodisetti S., Gadag S., Nayak U. Y., Govindan S., Raval K. (2020). Tailoring
Solulan C24 Based Niosomes for Transdermal Delivery of
Donepezil: In Vitro Characterization, Evaluation of PH Sensitivity,
and Microneedle-Assisted Ex Vivo Permeation Studies. J. Drug Delivery Sci. Technol..

[29] Saluja S., Kasha P. C., Paturi J., Anderson C., Morris R., Banga A. K. (2013). A Novel Electronic Skin Patch for
Delivery and Pharmacokinetic
Evaluation of Donepezil Following Transdermal Iontophoresis. Int. J. Pharm..

[30] Kale M., Kipping T., Banga A. K. (2020). Modulated Delivery
of Donepezil Using
a Combination of Skin Microporation and Iontophoresis. Int. J. Pharm..

[31] Atılay
Takmaz E., Yener G. (2021). Effective Factors on Iontophoretic
Transdermal Delivery of Memantine and Donepezil as Model Drugs. J. Drug Delivery Sci. Technol..

[32] Kodoth A. K., Ghate V. M., Lewis S. A., Prakash B., Badalamoole V. (2019). Pectin-Based
Silver Nanocomposite Film for Transdermal Delivery of Donepezil. Int. J. Biol. Macromol..

[33] Ertas B., Onay I. N., Yilmaz-Goler A. M., Karademir-Yilmaz B., Aslan I., Cam M. E. (2023). A Novel High-Efficiency
Transdermal
Patches for Combinational Therapy of Alzheimer’s Disease: Donepezil/Vitamin
B12-Loaded Nanofibers. J. Drug Delivery Sci.
Technol..

[34] Nieva-Echevarría B., Goicoechea E., Manzanos M. J., Guillén M. D. (2014). A Method
Based on 1H NMR Spectral Data Useful to Evaluate the Hydrolysis Level
in Complex Lipid Mixtures. Food Res. Int..

[35] Moreira D. N., Fresno N., Pérez-Fernández R., Frizzo C. P., Goya P., Marco C., Martins M. A. P., Elguero J. (2015). Brønsted Acid–Base Pairs of Drugs as Dual
Ionic Liquids: NMR Ionicity Studies. Tetrahedron.

[36] Berman H. M., Westbrook J., Feng Z., Gilliland G., Bhat T. N., Weissig H., Shindyalov I. N., Bourne P. E. (2000). The Protein Data Bank. Nucleic
Acids Res..

[37] Neese F. (2022). Software Update:
The ORCA Program System Version 5.0. WIREs Comput.
Mol. Sci..

[38] Bannwarth C., Ehlert S., Grimme S. (2019). GFN2-XTB An
Accurate and Broadly
Parametrized Self-Consistent Tight-Binding Quantum Chemical Method
with Multipole Electrostatics and Density-Dependent Dispersion Contributions. J. Chem. Theory Comput..

[39] Martínez L., Andrade R., Birgin E. G., Martínez J. M. (2009). PACKMOL:
A Package for Building Initial Configurations for Molecular Dynamics
Simulations. J. Comput. Chem..

[40] Wang J., Wolf R. M., Caldwell J. W., Kollman P. A., Case D. A. (2004). Development
and Testing of a General Amber Force Field. J. Comput. Chem..

[41] Jakalian A., Bush B. L., Jack D. B., Bayly C. I. (2000). Fast, Efficient
Generation of High-Quality Atomic Charges. AM1-BCC Model: I. Method. J. Comput. Chem..

[42] Berendsen H. J. C., Postma J. P. M., Van
Gunsteren W. F., Dinola A., Haak J. R. (1984). Molecular
Dynamics with Coupling to an External Bath. J. Chem. Phys..

[43] Pastor R. W., Brooks B. R., Szabo A. (1988). An Analysis of the Accuracy of Langevin
and Molecular Dynamics Algorithms. Mol. Phys..

[44] Darden T., York D., Pedersen L. (1993). Particle Mesh
Ewald: An N·log­(N)
Method for Ewald Sums in Large Systems. J. Chem.
Phys..

[45] Ryckaert J.-P., Ciccotti G., Berendsen H. J. C. (1977). Numerical
Integration of the Cartesian
Equations of Motion of a System with Constraints: Molecular Dynamics
of n-Alkanes. J. Comput. Phys..

[46] Costa P., Lobo J. M. S. (2001). Modeling and
Comparison of Dissolution Profiles. Eur. J.
Pharm. Sci..

[47] Ruela A. L. M., Santos M. G., Figueiredo E. C., Pereira G. R. (2014). LC-PDA and LC-MS
Studies of Donepezil Hydrochloride Degradation Behaviour in Forced
Stress Conditions. J. Braz. Chem. Soc..

[48] Committee for Medicinal Products for Human Use . International Conference of Harmonisation for Better Health. ICH Q2(R2); Guideline on Validation of Analytical Procedures; EMA/CHMP/ICH/82072/2006, 2006. https://www.ema.europa.eu/en/ich-q2r2-validation-analytical-procedures-scientific-guideline (accessed 2024–11–04).

[49] Aursand M., Grasdalen H. (1992). Interpretation of the 13C-NMR Spectra of Omega-3 Fatty
Acids and Lipid Extracted from the White Muscle of Atlantic Salmon
(Salmo Salar). Chem. Phys. Lipids.

[50] Kulkarni C. V., Wachter W., Iglesias-Salto G., Engelskirchen S., Ahualli S. (2011). Monoolein: A Magic Lipid?. Phys.
Chem. Chem. Phys..

[51] Gusntone F. D. (1991). 13C-NMR
Studies of Mono-, Di-and Tri-Acylglycerols Leading to Qualitative
and Semiquantitative Information about Mixtures of These Glycerol
Esters. Chem. Phys. Lipids.

[52] Fattori, J. ; Rodrigues, F. H. S. ; Pontes, J. G. M. ; Espíndola, A. P. ; Tasic, L. Monitoring Intermolecular and Intramolecular Interactions by NMR Spectroscopy. In Applications of NMR Spectroscopy; ur-Rahman, A. , Choudhary, I. , Eds.; Bentham Science Publishers Ltd, 2015; Vol. 3, pp 180–266.

[53] De
Roo J., Ibáñez M., Geiregat P., Nedelcu G., Walravens W., Maes J., Martins J. C., Van Driessche I., Kovalenko M. V., Hens Z. (2016). Highly Dynamic Ligand Binding and
Light Absorption Coefficient of Cesium Lead Bromide Perovskite Nanocrystals. ACS Nano.

[54] Astolfi P., Giorgini E., Perinelli D. R., Vita F., Adamo F. C., Logrippo S., Parlapiano M., Bonacucina G., Pucciarelli S., Francescangeli O., Vaccari L., Pisani M. (2021). Cubic and
Hexagonal Mesophases for Protein Encapsulation: Structural Effects
of Insulin Confinement. Langmuir.

[55] Ramos L. K. S., Santana H. V., Marques V. C., Leão L. G., Fernandes G. R. R., De Souza Filho J. D., Pereira G. R., De Souza J., Ruela A. L. M. (2025). Phase Transition
Behavior, Mucoadhesion, and Modified
Release Properties from in Situ Gelling Monoglycerides-Based Formulations
with Acyclovir. J. Drug Delivery Sci. Technol..

[56] Korting H. C., Megele M., Mehringer L., Vieluf D., Zienicke H., Hamm G., Braun-Falco O. (1991). Influence
of Skin Cleansing Preparation
Acidity on Skin Surface Properties. Int. J.
Cosmet. Sci..

[57] Ruela A. L. M., Perissinato A. G., Lino M. E., Mudrik P. S., Pereira G. R., Pereira G. R. (2016). Evaluation
of Skin Absorption of
Drugs from Topical and Transdermal Formulations. Brazilian J. Pharm. Sci..

[58] Mendes I. T., Ruela A. L. M., Carvalho F. C., Freitas J. T. J., Bonfilio R., Pereira G. R. (2019). Development and
Characterization of Nanostructured
Lipid Carrier-Based Gels for the Transdermal Delivery of Donepezil. Colloids Surf., B.

[59] Elgindy N. A., Mehanna M. M., Mohyeldin S. M. (2016). Self-Assembled
Nano-Architecture
Liquid Crystalline Particles as a Promising Carrier for Progesterone
Transdermal Delivery. Int. J. Pharm..

[60] Siekmann B., Bunjes H., Koch M. H. J., Westesen K. (2002). Preparation and Structural
Investigations of Colloidal Dispersions Prepared from Cubic Monoglyceride–Water
Phases. Int. J. Pharm..

[61] Lambers H., Piessens S., Bloem A., Pronk H., Finkel P. (2006). Natural Skin
Surface PH Is on Average below 5, Which Is Beneficial for Its Resident
Flora. Int. J. Cosmet. Sci..

[62] Schmid-Wendtner M.-H., Korting H. C. (2006). The PH of the Skin
Surface and Its Impact on the Barrier
Function. Skin Pharmacol. Physiol..

[63] Dick I. P., Scott R. C. (1992). Pig Ear Skin as
an In-Vitro Model for Human Skin Permeability. J. Pharm. Pharmacol..

[64] Sekkat N., Kalia Y. N., Guy R. H. (2002). Biophysical
Study of Porcine Ear
Skin In Vitro and Its Comparison to Human Skin In Vivo. J. Pharm. Sci..

[65] Jacobi U., Kaiser M., Toll R., Mangelsdorf S., Audring H., Otberg N., Sterry W., Lademann J. (2007). Porcine Ear
Skin: An in Vitro Model for Human Skin. Ski.
Res. Technol..

[66] Rattanapak T., Young K., Rades T., Hook S. (2012). Comparative Study of
Liposomes, Transfersomes, Ethosomes and Cubosomes for Transcutaneous
Immunisation: Characterisation and in Vitro Skin Penetration. J. Pharm. Pharmacol..

[67] Guy R. H., Hadgraft J. (1987). Transdermal Drug Delivery:
A Perspective. J. Controlled Release.

[68] Rowland, M. ; Tozer, T. . In Clinical Pharmacokinetics and Pharmacodynamics: Concepts and Applications, 4 ed.; Rowland, M. , Tozer, T. N. , Eds.; Lippincott Williams & Wilkins: Philadelphia, PA, United States, 2011.

[69] Rogers S. L.., Friedhoff L. T. (1998). Pharmacokinetic and Pharmacodynamic Profile of Donepezil
HCl Following Single Oral Doses. Br. J. Clin.
Pharmacol..

[70] Belade E., Armand L., Martinon L., Kheuang L., Fleury-Feith J., Baeza-Squiban A., Lanone S., Billon-Galland M.-A., Pairon J.-C., Boczkowski J. (2012). A Comparative
Transmission Electron
Microscopy Study of Titanium Dioxide and Carbon Black Nanoparticles
Uptake in Human Lung Epithelial and Fibroblast Cell Lines. Toxicol. Vitr..

[71] Zhang M., Zhao F., Zhang X., Brouwer L. A., Burgess J. K., Harmsen M. C. (2023). Fibroblasts Alter the Physical Properties
of Dermal
ECM-Derived Hydrogels to Create a pro-Angiogenic Microenvironment. Mater. Today Bio.

[72] Bahari N., Hashim N., Abdan K., Akim A. M., Maringgal B., Al-Shdifat L. (2025). Green-Synthesised Silver and Zinc Oxide Nanoparticles
from Stingless Bee Honey: Morphological Characterisation, Antimicrobial
Action, and Cytotoxic Assessment. Chemosphere.

[73] Kumar C. G., Poornachandra Y. (2015). Biodirected
Synthesis of Miconazole-Conjugated Bacterial
Silver Nanoparticles and Their Application as Antifungal Agents and
Drug Delivery Vehicles. Colloids Surf., B.

[74] Suthar T., Maurya R., Sonwani A., Upadhayay P., Datusalia A. K., Naqvi S., Jain K. (2026). Nose-to-Brain
Delivery
of Donepezil Hydrochloride via Oleic Acid-Conjugated PAMAM G4 Dendrimers
for the Treatment of Alzheimer’s-Like Dementia. Mol. Pharm..

