# Supplementary material for: Modulation of Donepezil Release Through Hydrophobic Ion- Pairing with Oleic Acid in Liquid Crystalline Nanodispersions for Transdermal Delivery
Source: ACS Omega. 2026 Jul 17;11(33):49938–53. doi: 10.1021/acsomega.6c04903 (PMC13520556; doi:10.1021/acsomega.6c04903)
Supplement: Supplementary file 1 [file ao6c04903_si_002.pdf]

Supplementary Material

for

Modulation of donepezil release through hydrophobic ion-pairing with oleic acid in liquid crystalline nanodispersions for transdermal delivery

Priscila Pires da Silva[a]; Victor Cavalcanti Marques[a]; Lorena Kelly Santiago Ramos[a]; José Dias de Souza Filho[a]; Renato Ferreira de Freitas[a]; Marina Guimarães Carvalho Machado[a]; Marcus Vinícius Cangussu Cardoso[b]; Flávia de Souza Marques[c]; Paula Melo de Abreu Vieira[c]; Leonardo da Silva Araújo[d]; Jaqueline dos Santos Soares[d]; Jacqueline de Souza[a]; André Luís Morais Ruela[a,*]

[a] Graduate Program of Pharmaceutical Sciences, School of Pharmacy, Federal University of Ouro Preto, UFOP, Minas Gerais, Brazil.

[b] Graduate Program in Biotechnology, Institute of Exact and Biological Sciences, Federal University of Ouro Preto, UFOP, Minas Gerais, Brazil.

[c] Graduate Program in Biological Sciences, Institute of Exact and Biological Sciences, Federal University of Ouro Preto, UFOP, Minas Gerais, Brazil.

[d] Department of Physics, Institute of Exact and Biological Sciences, Federal University of Ouro Preto, UFOP, Minas Gerais, Brazil.

* Correspondence to André L.M. Ruela (Telephone: 55-31-3599-1040; E-mail address: andre.ruela@ufop.edu.br)

Nuclear magnetic resonance (NMR)

Based on literature reports, we analyzed the NMR spectra of donepezil (Figures S1 and S2) [1,2] as well as the spectra of the lipids used in the formulations [3–6].

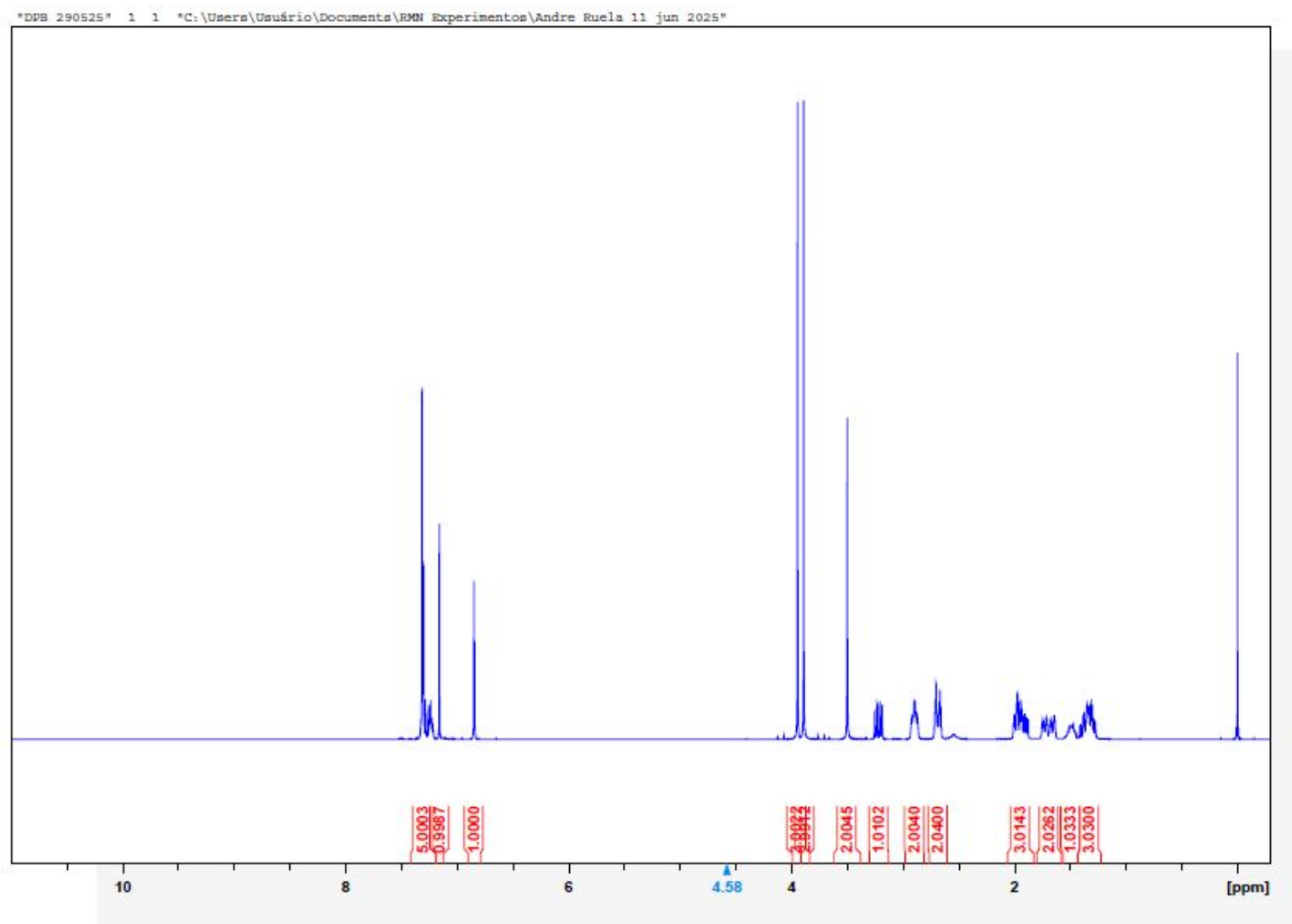

Figure S1. ^{1}H NMR spectrum of donepezil (400 MHz, chloroform-d) δ 7.34–7.20 (m, 5H), 7.16 (s, 1H), 6.85 (s, 1H), 3.94 (s, 3H), 3.89 (s, 3H), 3.50 (s, 2H), 3.22 (q, J = 8.2 Hz, 4H), 2.97–2.81 (m, 2H), 2.75–2.63 (m, 2H), 2.04–1.85 (m, 3H), 1.73–1.61 (m, 2H), 1.56–1.43 (m, 1H), 1.43–1.24 (m, 3H).

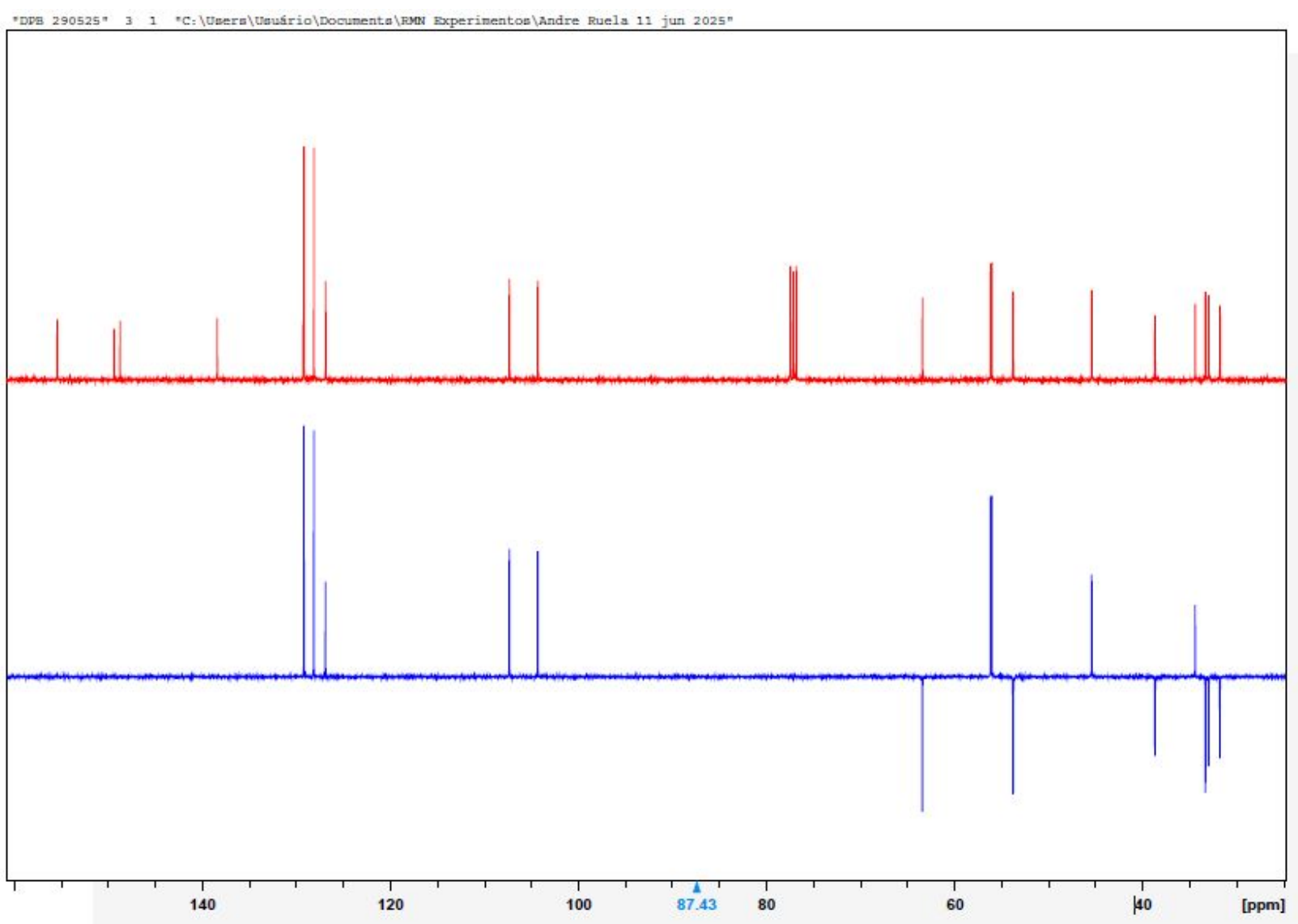

Figure S2. ^{13}C NMR spectrum of donepezil (100 MHz, chloroform-d; red line) δ: 207.72, 155.40, 149.37, 148.72, 138.39, 129.26, 129.18, 128.09, 126.87, 107.33, 104.31, 63.40, 56.16, 56.03, 53.75, 53.73, 45.42, 38.67, 34.41, 33.30, 32.98, 31.76. The blue line corresponds to the ^{13}C NMR DEPT-135 subspectrum of donepezil (100 MHz,

chloroform-d) δ: 138.40, 129.18, 128.93, 128.09, 126.87, 107.34, 104.32, 63.41 (CH_2), 56.16, 56.03, 53.76 (CH_2), 53.74 (CH_2), 45.42, 38.68 (CH_2), 34.42, 33.30 (CH_2), 32.99 (CH_2), 31.76 (CH_2). All methylene (CH_2) carbons appear in the negative phase.

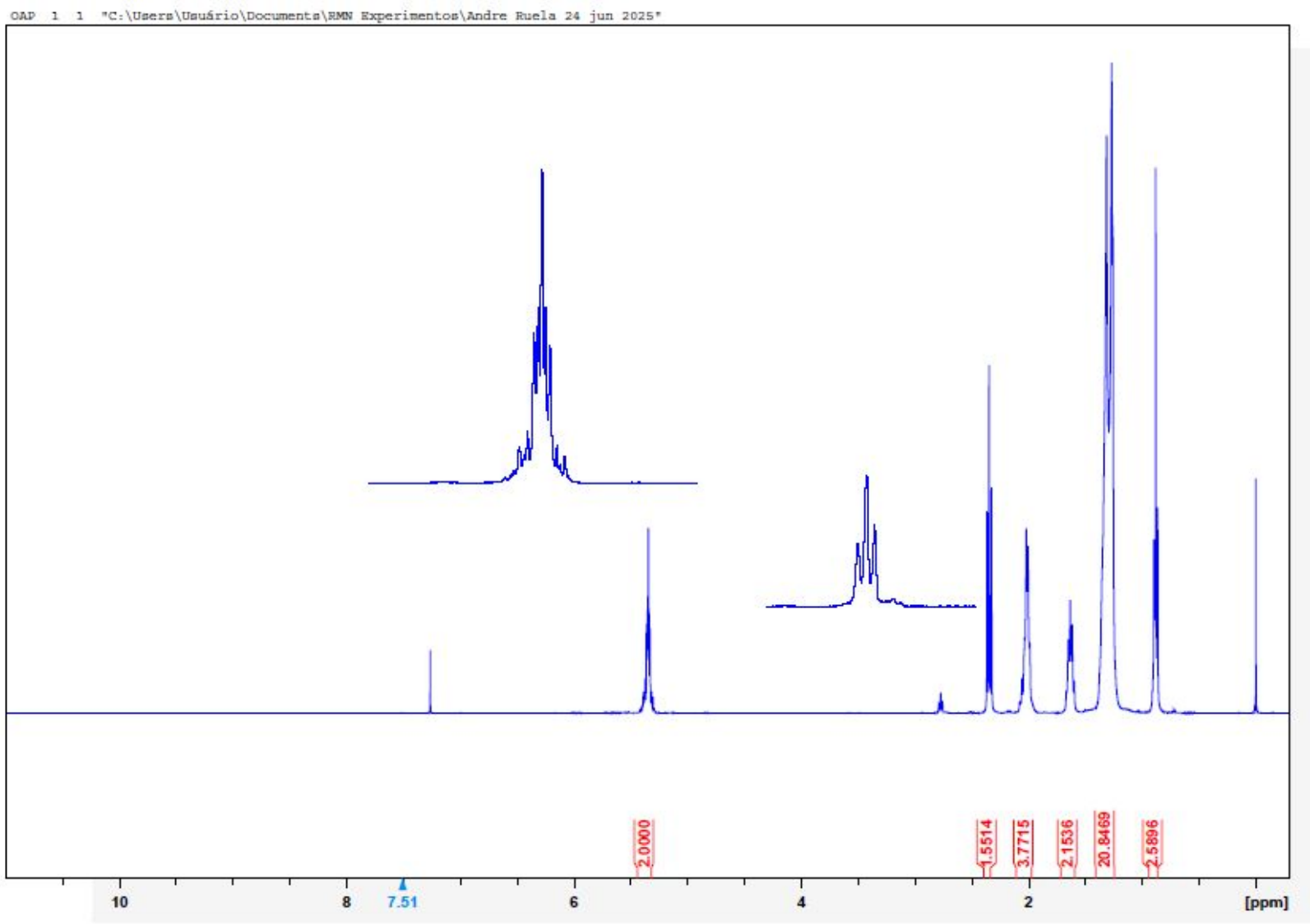

Figure S3. ^{1}H NMR spectrum of oleic acid (400 MHz, chloroform-d) δ 5.44–5.27 (m, 2H), 2.77 (t, J = 6.5 Hz, trace signal from residual polyunsaturated fatty acids), 2.34 (t, J = 7.7 Hz, 2H), 2.12–1.92 (m, 4H), 1.72–1.54 (m, 2H), 1.50–1.07 (m, 20H), 0.93–0.83 (m, 3H). The characteristic resonances highlighted in the figure are located at around 5.44–5.27 and 2.77 ppm.

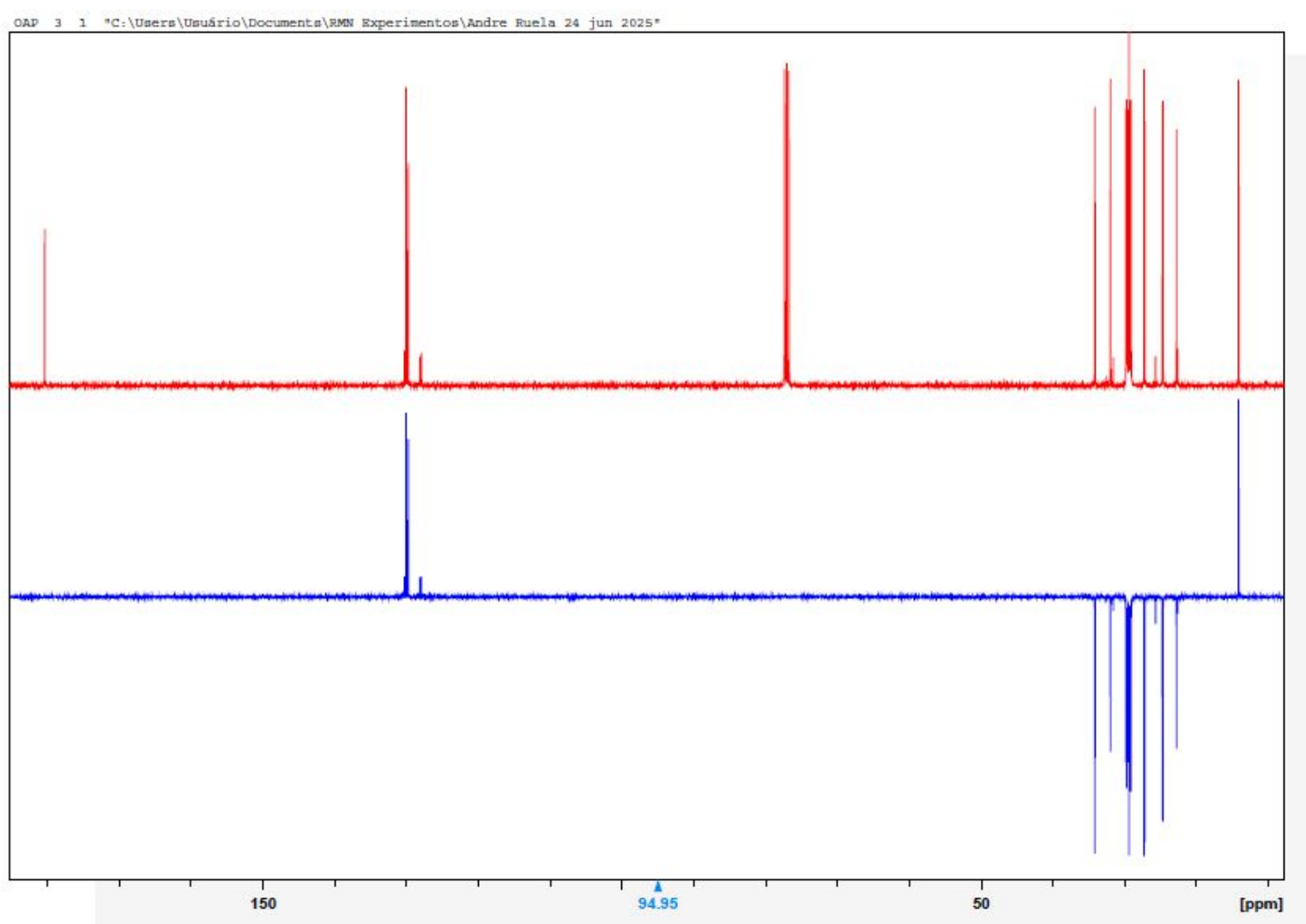

Figure S4. ^{13}C NMR spectrum of oleic acid (100 MHz, $CDCl_3$; red line). δ: 180.34, 130.24, 130.03, 129.74, 128.05, 127.94, 34.11, 31.93, 29.79, 29.70, 29.55, 29.34, 29.16, 29.09, 29.05, 27.23, 27.17, 24.68, 22.70, 14.12. Minor olefinic signals at 130.24, 128.05, and 127.94 ppm are consistent with traces of additional unsaturated fatty

acids. Additional minor methylene (CH_2) signals were also detected at 31.56, 26.62, 29.47, 29.27, and 22.60 ppm. The blue line corresponds to the DEPT-135 subspectrum of oleic acid (100 MHz, $CDCl_3$). δ: 130.04, 129.74, 34.11 (CH_2), 31.93 (CH_2), 29.79 (CH_2), 29.70 (CH_2), 29.55 (CH_2), 29.35 (CH_2), 29.16 (CH_2), 29.09 (CH_2), 29.06 (CH_2), 27.24 (CH_2), 27.17 (CH_2), 24.68 (CH_2), 22.71 (CH_2), 14.12 (CH_3). As expected, the carboxyl carbon (~180 ppm) is absent, and the methylene (CH_2) carbons appear in the negative phase. The additional minor methylene (CH_2) signals at 31.56, 26.62, 29.47, 29.27, and 22.60 ppm also appear in the negative phase.

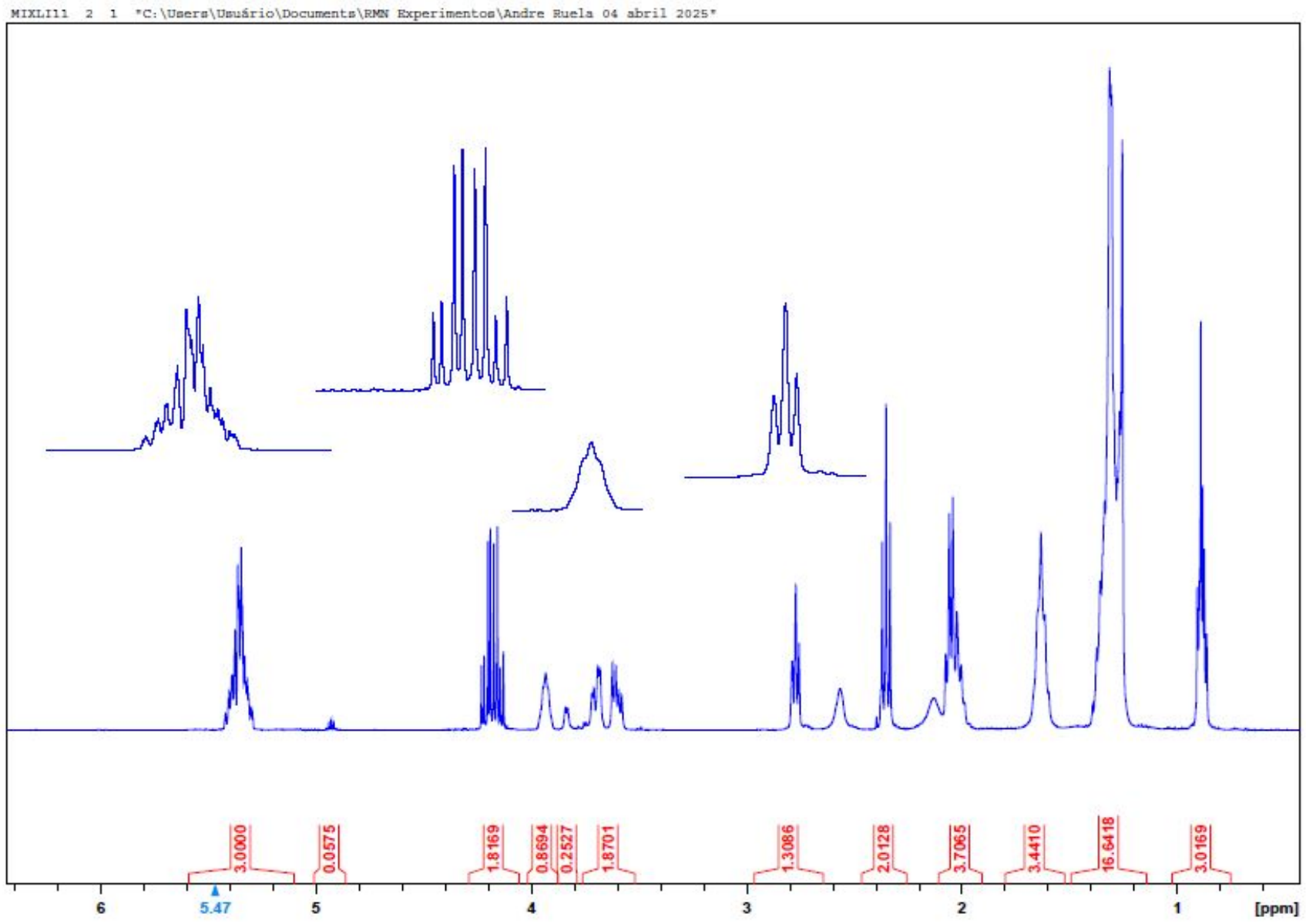

Figure S5. ^{1}H NMR spectrum of monoglycerides (400 MHz, chloroform-d) δ 5.45–5.28 (m, 3H), 4.18 (ddd, 2H, J ≈ 5.5 Hz), 3.97–3.89 (m, 1H), 3.65 (ddd, 2H, J ≈ 5.0 Hz), 2.77 (t, 1H, J ≈ 6.0 Hz), 2.35 (t, 2H, J = 7.7 Hz), 2.09–1.97 (m, 4H), 1.70–1.57 (m, 3H), 1.42–1.21 (m, 17H), 0.93–0.84 (m, 3H). Trace signals from residual 2-monoglycerides were observed at 4.97–4.90 (m) and 3.87–3.80 (m). The characteristic resonances highlighted in the figure are located at around 5.45–5.28, 4.18, 3.97–3.89, and 2.77 ppm.

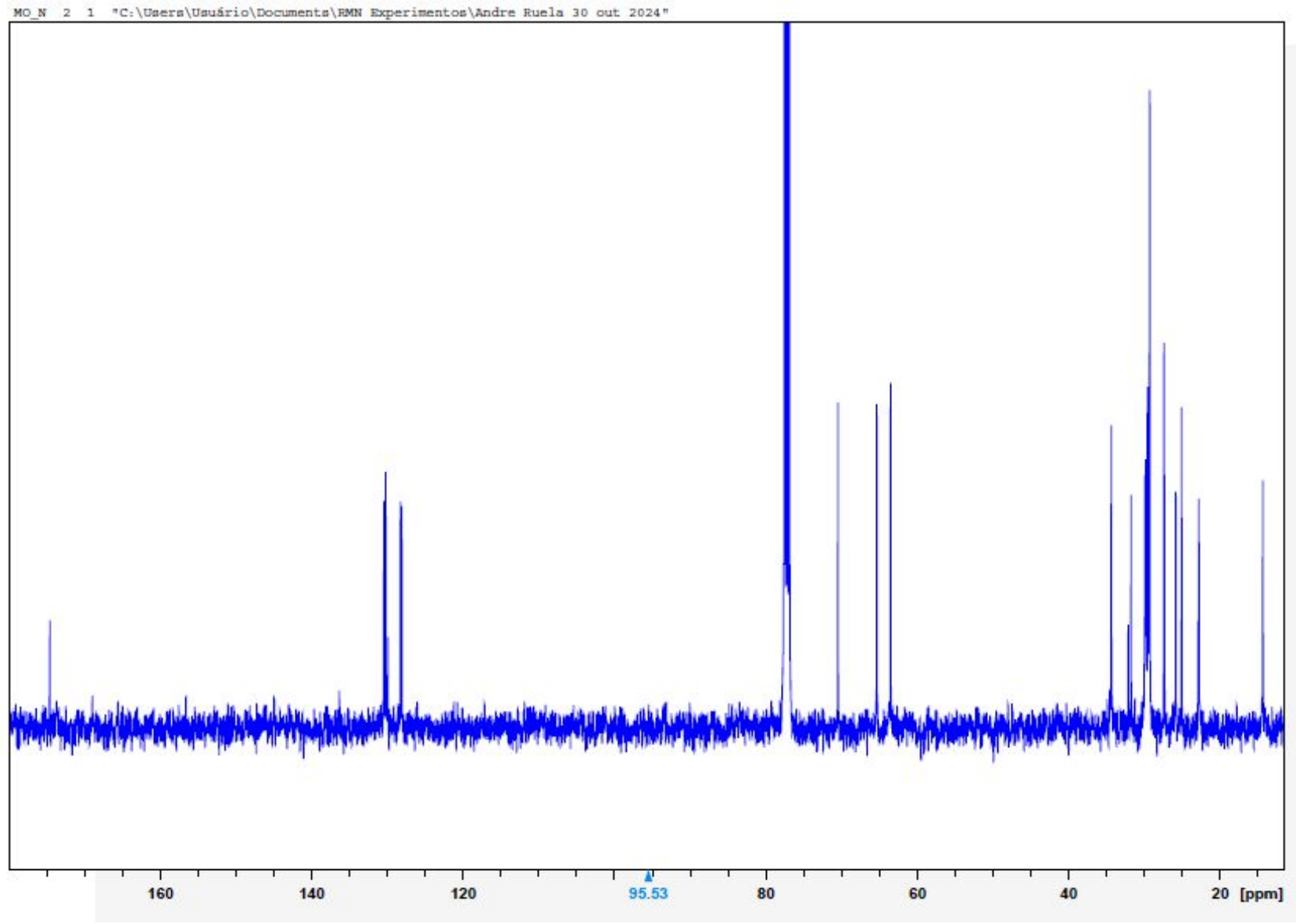

Figure S6. ^{13}C NMR spectrum of monoglycerides (100 MHz, $CDCl_3$). δ: 174.58, 130.44, 130.22, 128.27, 128.08, 70.45, 65.35, 63.52, 34.33, 32.11, 31.72, 29.89, 29.79, 29.54, 29.35, 29.29, 27.38, 25.82, 25.08, 22.78, 14.29. Minor olefinic signals at 129.91, 29.96, 29.73, 29.66, 29.46, 22.89 ppm are consistent with traces of additional unsaturated fatty acids.

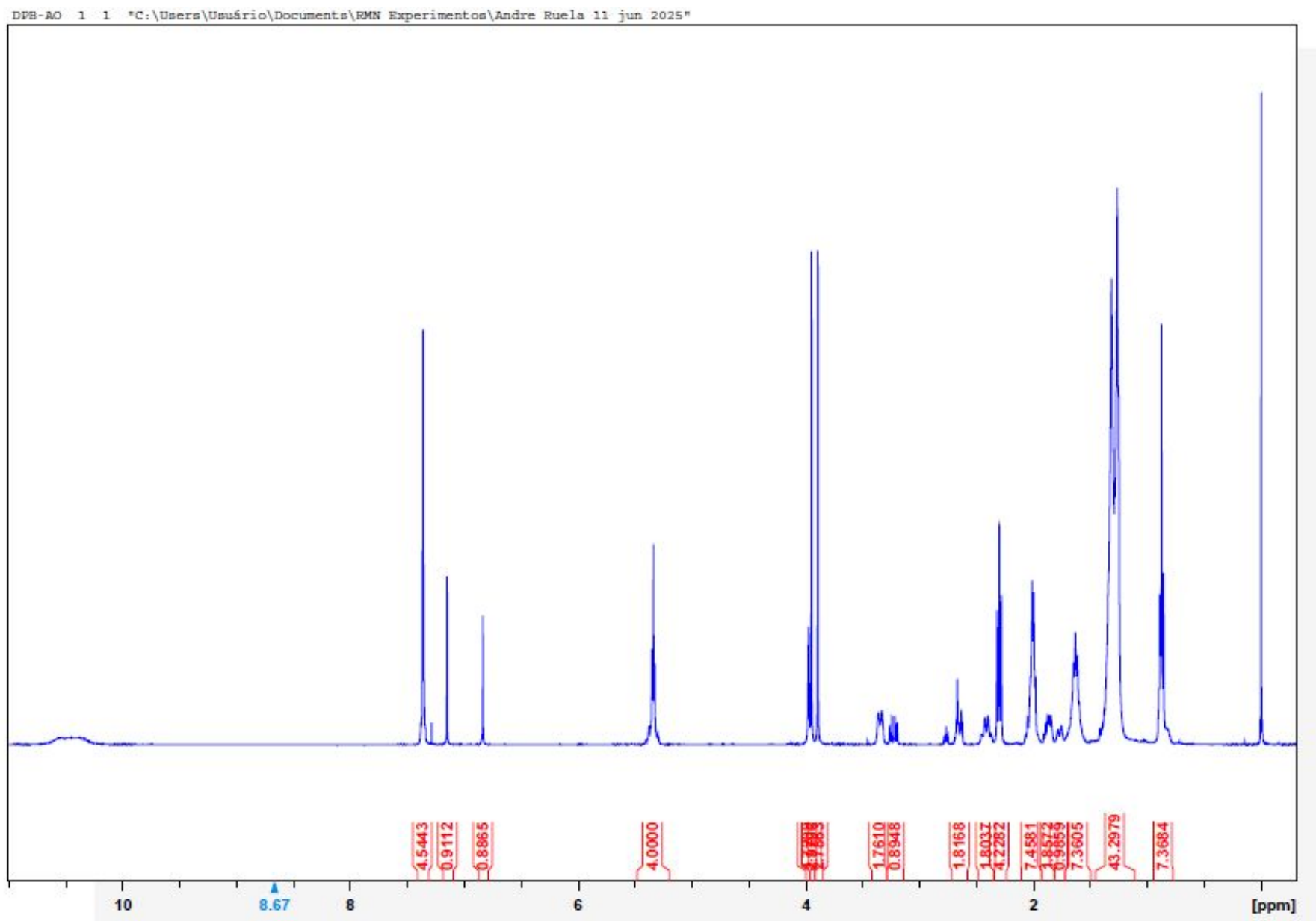

Figure S7. ^{1}H NMR spectrum of the donepezil–oleic acid ion pair at a molar ratio of 1:2 (400 MHz, chloroform-d): δ 7.36 (br s, 5H), 7.15 (s, 1H), 6.84 (s, 1H), 5.42–5.27 (m, 4H), 3.98 (br s, 2H), 3.95 (s, 3H), 3.89 (s, 3H), 3.42–3.16 (m, 3H), 2.72–2.60 (m, 2H), 2.41 (br q, J ≈ 11.0 Hz, 2H), 2.30 (t, J = 7.5 Hz, 4H), 2.10–1.93 (m, 7H), 1.93–1.82 (m, 2H), 1.81–1.73 (m, 1H), 1.72–1.53 (m, 7H), 1.45–1.15 (m, 43H), 0.94–0.78 (m, 7H).

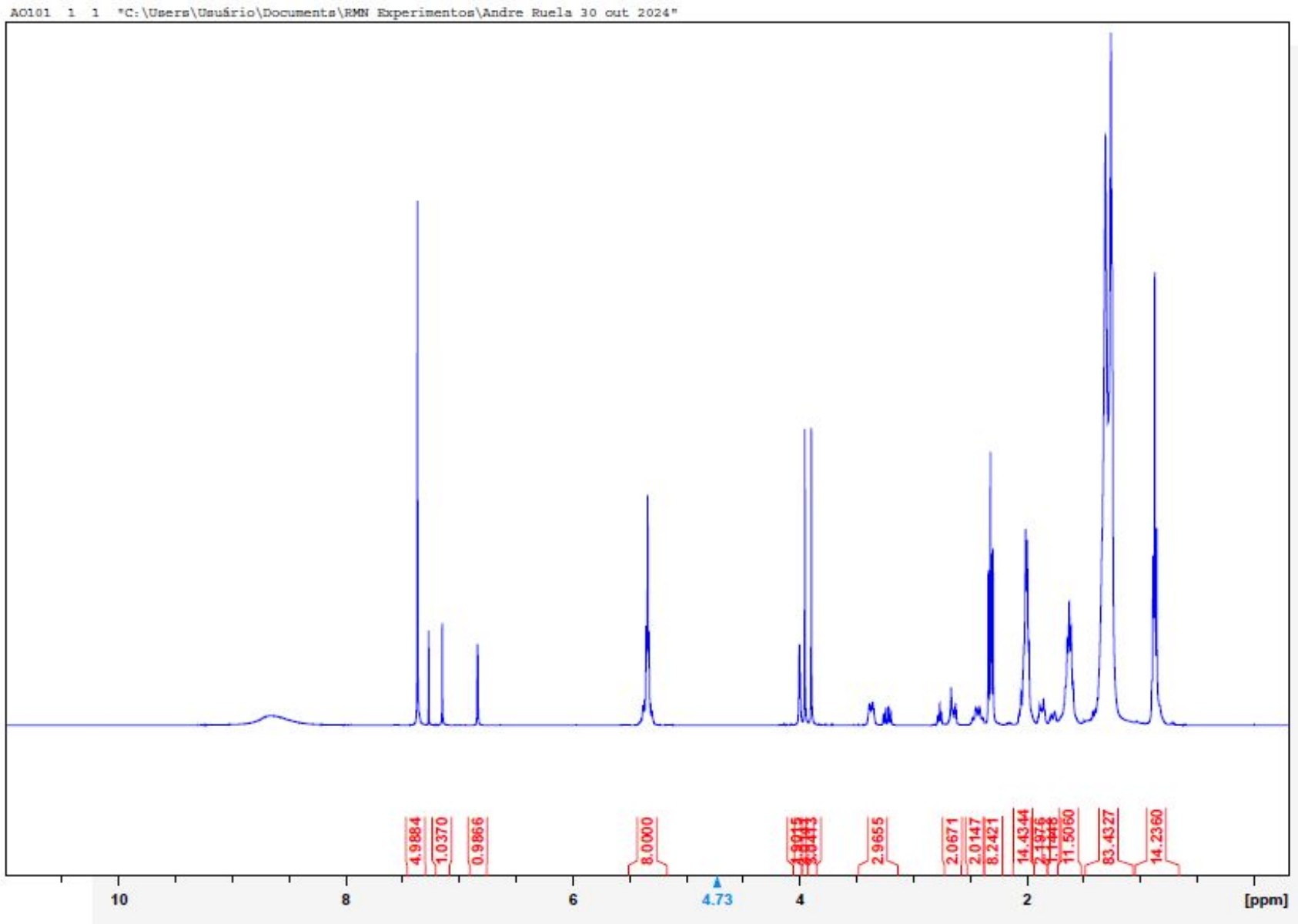

Figure S8. ¹H NMR spectrum of the donepezil–oleic acid ion pair at a molar ratio of 1:4 (400 MHz, chloroform-d): δ 7.37 (br s, 5H), 7.15 (s, 1H), 6.84 (s, 1H), 5.45–5.24 (m, 8H), 4.01 (br s, 2H), 3.96 (s, 3H), 3.90 (s, 3H), 3.48–3.14 (m, 3H), 2.73–2.58 (m, 2H), 2.44 (br q, J ≈ 11.2 Hz, 2H), 2.32 (t, J = 7.5 Hz, 8H), 2.13–1.92 (m, 14H), 1.93–1.82 (m, 2H), 1.82–1.74 (m, 1H), 1.73–1.52 (m, 12H), 1.52–0.97 (m, 83H), 0.99–0.70 (m, 14H).

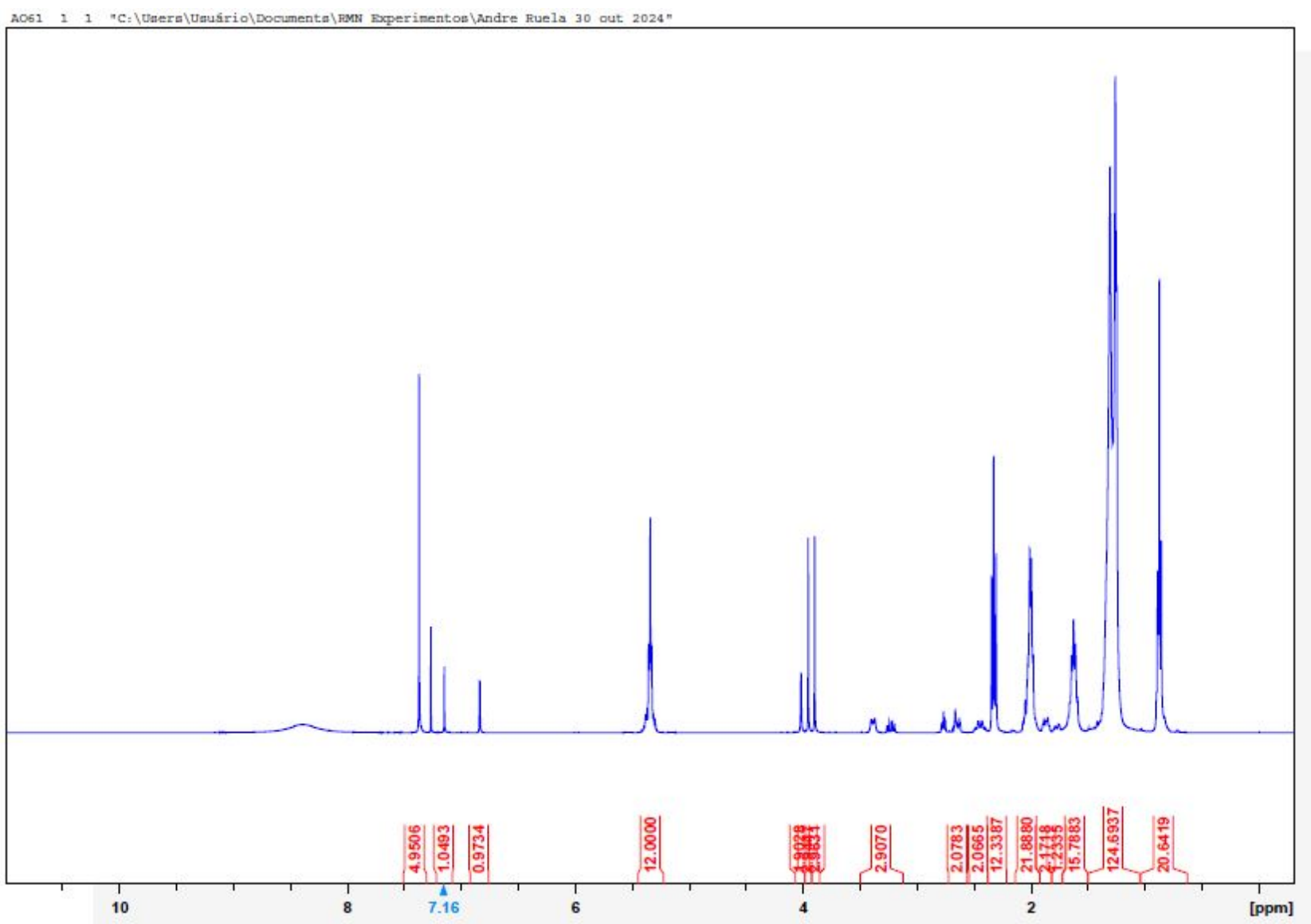

Figure S9. ¹H NMR spectrum of the donepezil–oleic acid ion pair at a molar ratio of 1:6 (400 MHz, chloroform-d): δ 7.37 (s, 5H), 7.15 (s, 1H), 6.84 (s, 1H), 5.45–5.26 (m, 12H), 4.02 (s, 2H), 3.96 (s, 3H), 3.90 (s, 3H), 3.47–3.15 (m, 3H), 2.72–2.57 (m, 2H), 2.55–2.38 (m, 2H), 2.34 (t, J = 7.5 Hz, 12H), 2.12–1.93 (m, 22H), 1.92–1.82 (m, 2H), 1.82–1.73 (m, 1H), 1.73–1.50 (m, 16H), 1.51–1.04 (m, 125H), 1.04–0.64 (m, 21H).

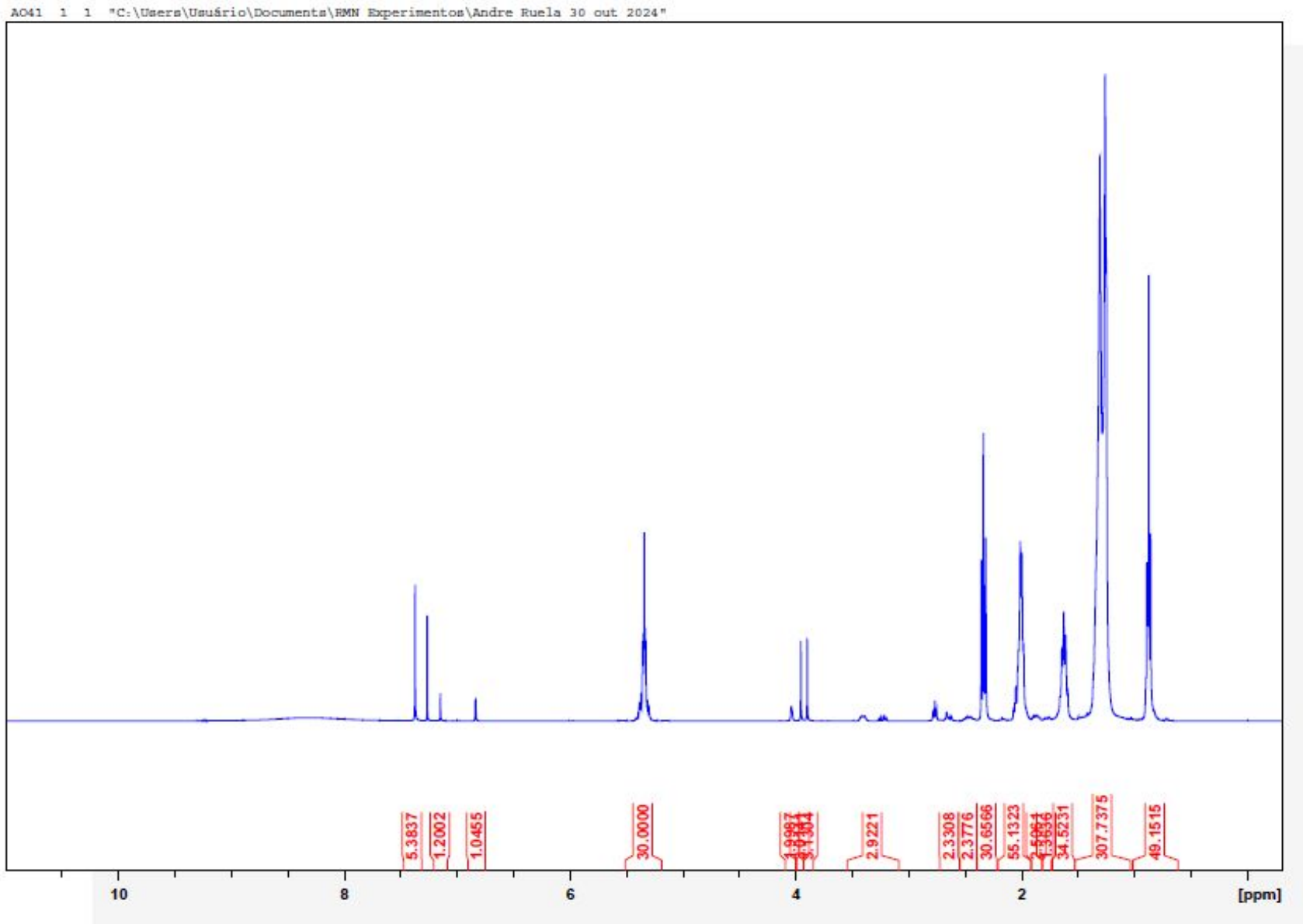

Figure S10. ^{1}H NMR spectrum of the donepezil–oleic acid ion pair at a molar ratio of 1:15 (400 MHz, chloroform-d): δ 7.37 (s, 5H), 7.15 (s, 1H), 6.84 (s, 1H), 5.46–5.20 (m, 30H), 4.04 (s, 2H), 3.96 (s, 3H), 3.90 (s, 3H), 3.50–3.15 (m, 3H), 2.72–2.56 (m, 2H), 2.55–2.40 (m, 2H), 2.34 (t, J = 7.5 Hz, 31H), 2.21–1.93 (m, 55H), 1.92–1.82 (m, 2H), 1.81–1.74 (m, 1H), 1.73–1.53 (m, 35H), 1.53–0.98 (m, 308H), 0.98–0.68 (m, 49H).

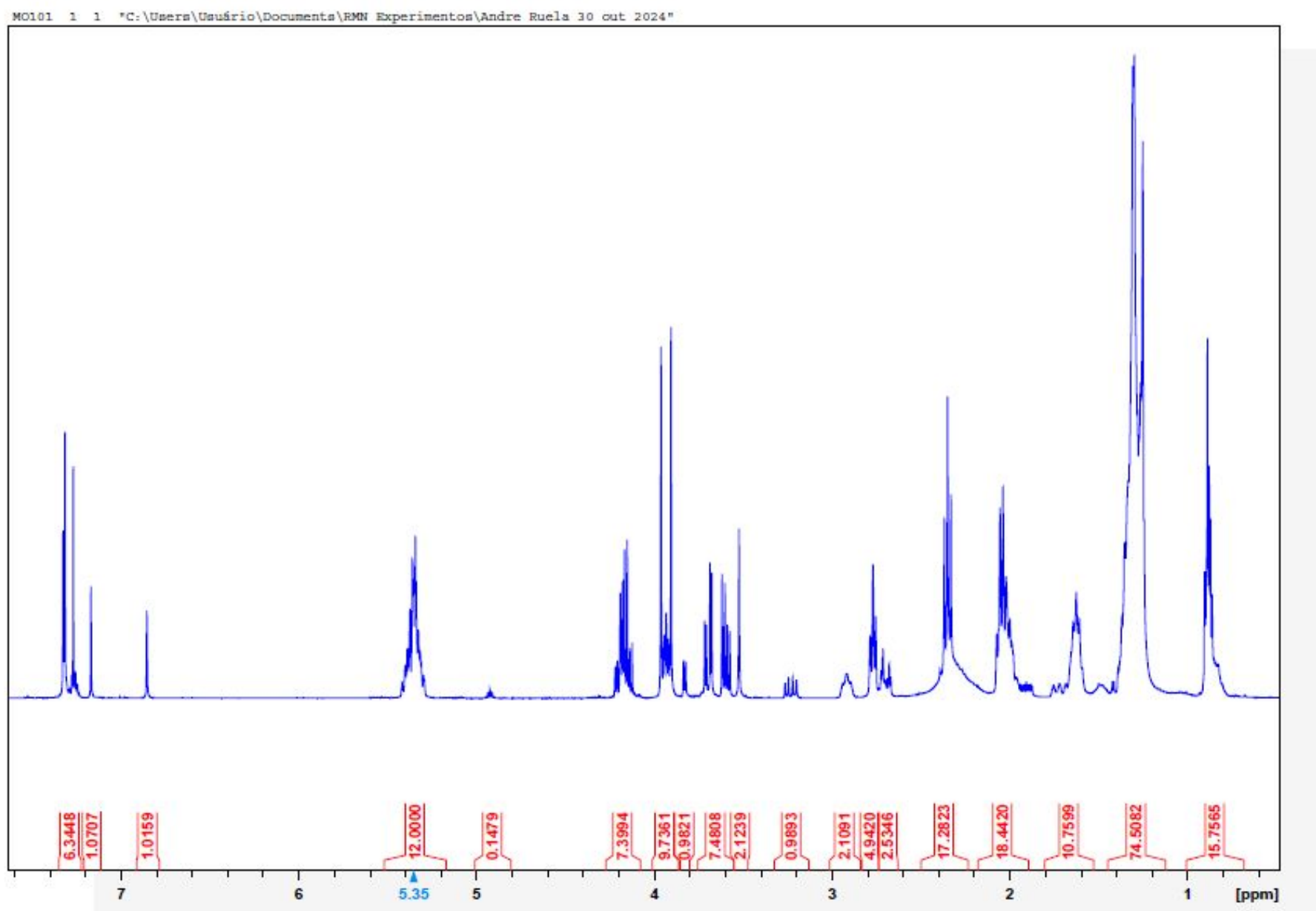

Figure S11. ^{1}H NMR spectrum of a donepezil-unsaturated monogycerides mixture at a molar ratio of 1:6 (400 MHz, $CDCl_3$) δ 7.35-7.22 (m, 6H), 7.17 (s, 1H), 6.85 (s, 1H), 5.44-5.24 (m, 12H), 4.17 (ddd, 7H), , 4.01-3.86 (m, 10H) 3.83 (m, 1H), 3.65 (ddd, 8H, J ~ 5.0 Hz), 3.53 (2H, s), 3.23 (dd, 1H, J ~ 8.0 Hz), 2.93-2.84 (m, 2H), 2.76

(5H, t, J ~ 6.0 Hz), 2.74-2,63 (m, 2H), 2.35 (t, 17H, J ~ 7.5 Hz), 2.12-1.89 (m, 18H), 1.80-1.54 (m, 11H), 1.45-1.11 (m, 75H), 0.98-0.71 (m, 16H). Trace signals from residual 2-monoglycerides were observed at 4.97-4.86 (m).

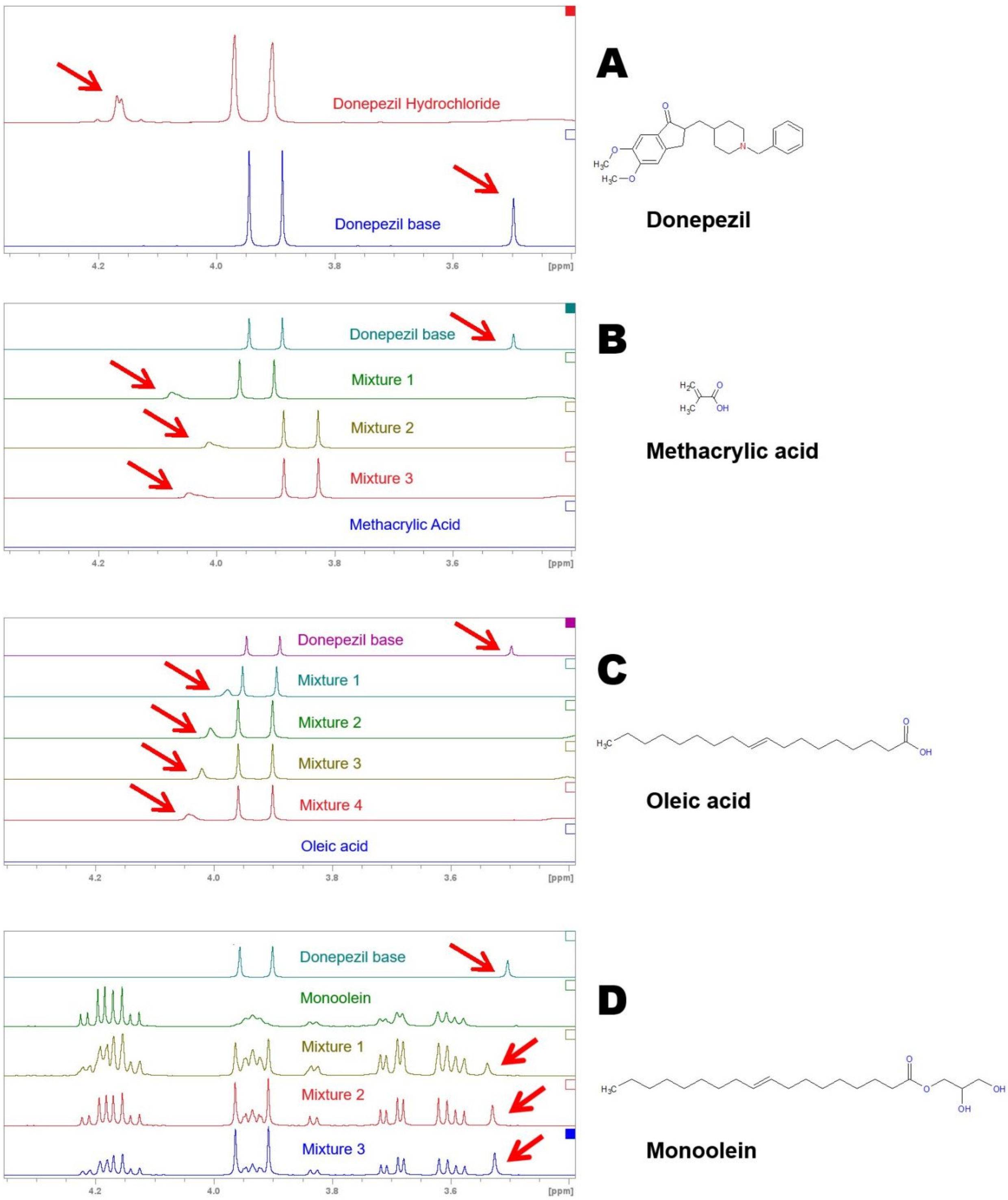

Figure S12. ^{1}H NMR spectra of donepezil and its mixtures with methacrylic acid, oleic acid, and unsaturated monoglycerides (400 MHz, $CDCl_3$). (A) Donepezil base and donepezil hydrochloride. (B) Mixtures of donepezil

with methacrylic acid at molar ratios of 1:2 (Mixture 2), 1:4 (Mixture 3), and 1:8 (Mixture 1); with oleic acid at molar ratios of 1:2 (Mixture 1), 1:4 (Mixture 2), 1:6 (Mixture 3), and 1:15 (Mixture 4); and with unsaturated monoglycerides (predominantly monoolein) at molar ratios of 1:6 (Mixture 3), 1:8 (Mixture 2), and 1:18 (Mixture 1).

Table S1. ^{1}H NMR chemical shifts, measured relative to tetramethylsilane in deuterated $CDCl_3$ at 400 MHz, used to calculate the percentage of free base (B%). The chemical shifts were determined from the benzylic methylene protons of the N-benzyl substituent of donepezil.

Sample	Chemical shifts (ppm)	Degree of ionicity (%)*
Donepezil free base	3.50	0.0
Donepezil hydrochloride	4.20	100.0
Donepezil and oleic acid at molar ratio of 1:2	3.98	68.6
Donepezil and oleic acid at molar ratio of 1:4	4.01	72.9
Donepezil and oleic acid at molar ratio of 1:6	4.02	74.3
Donepezil and oleic acid at molar ratio of 1:15	4.04	77.1
Donepezil and methacrylic acid at molar ratio of 1:2	4.01	72.9
Donepezil and methacrylic acid at molar ratio of 1:4	4.04	77.1
Donepezil and methacrylic acid at molar ratio of 1:8	4.07	81.4
Donepezil and unsaturated monoglycerides (predominantly C18 monoglycerides) at molar ratio of 1:6	3.53	4.3**
Donepezil and unsaturated monoglycerides (predominantly C18 monoglycerides) at molar ratio of 1:8	3.53	4.3**
Donepezil and unsaturated monoglycerides (predominantly C18 monoglycerides) at molar ratio of 1:18	3.54	5.7**

* According to Moreira and coworkers (2015) [7].

** Possibly associated with the presence of free fatty acids (acid value = 1.2 mg KOH/g, according to the manufacturer's certificate of analysis).

Table S2. Polarized light microscopy analysis of donepezil–oleic acid mixtures prepared at different molar ratios.

Sample	Polarized light microscopy (Magnification 40x)		Without polarization (Magnification 40x)
Mix 1: Donepezil and oleic acid at molar ratio of 1:1	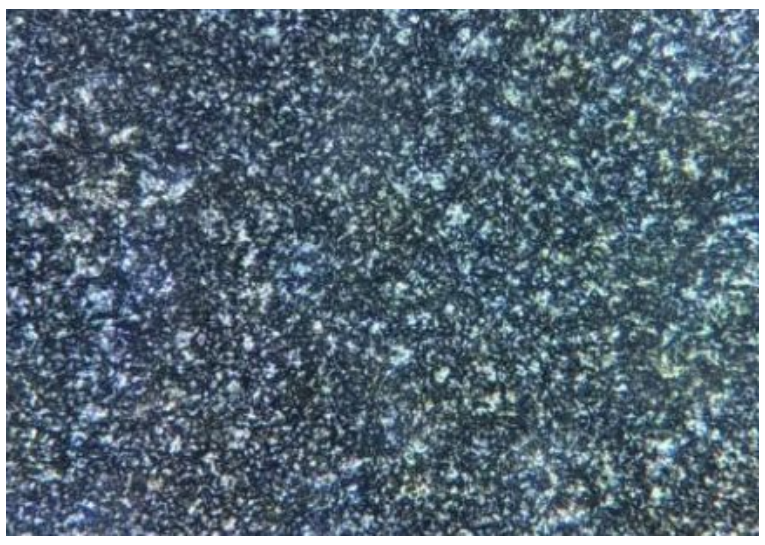	Anisotropy	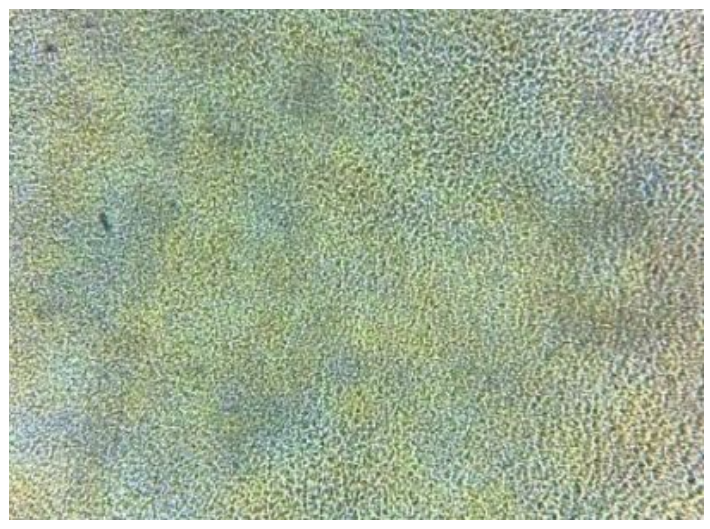
Mix 2: Donepezil and oleic acid at molar ratio of 1:2	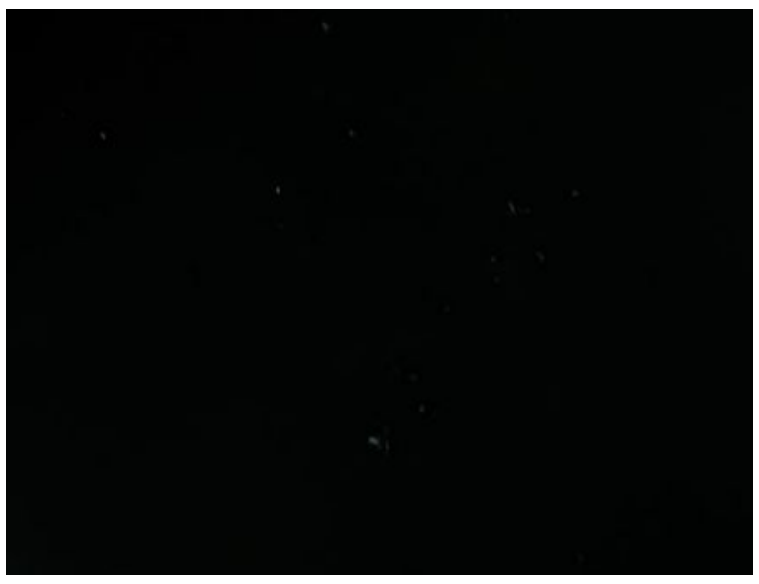	Isotropy	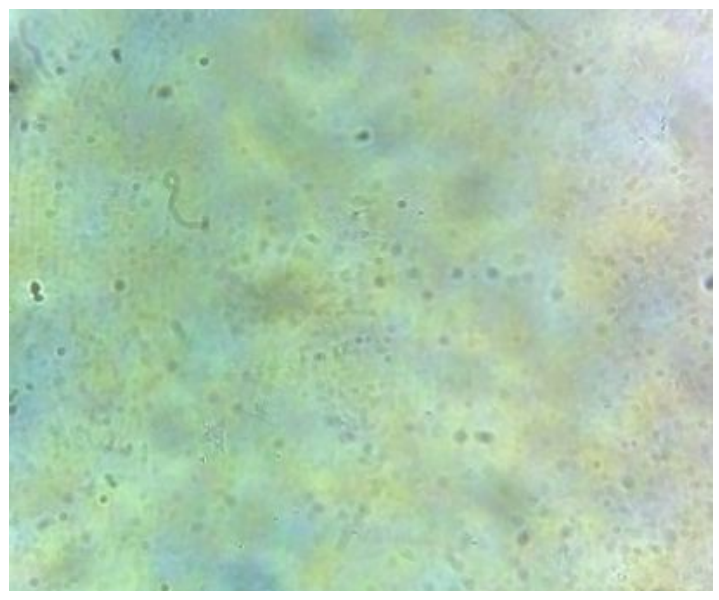
Mix 3: Donepezil and oleic acid at molar ratio of 1:3	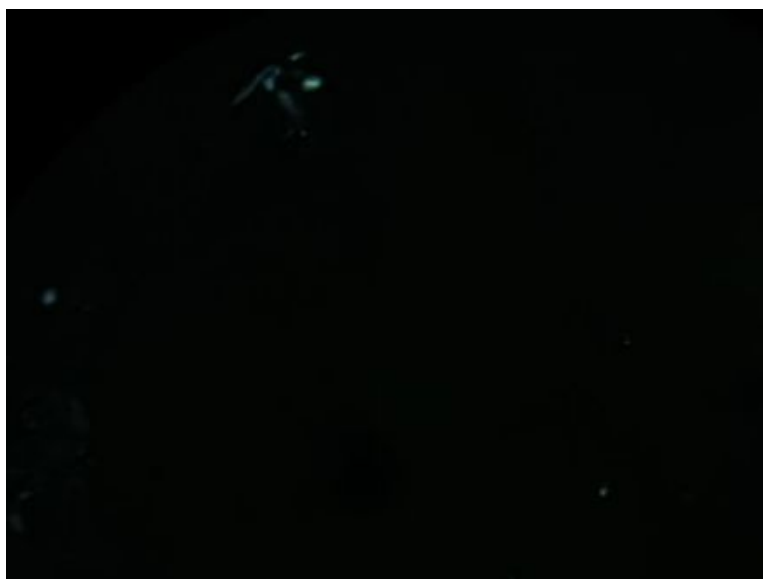	Isotropy	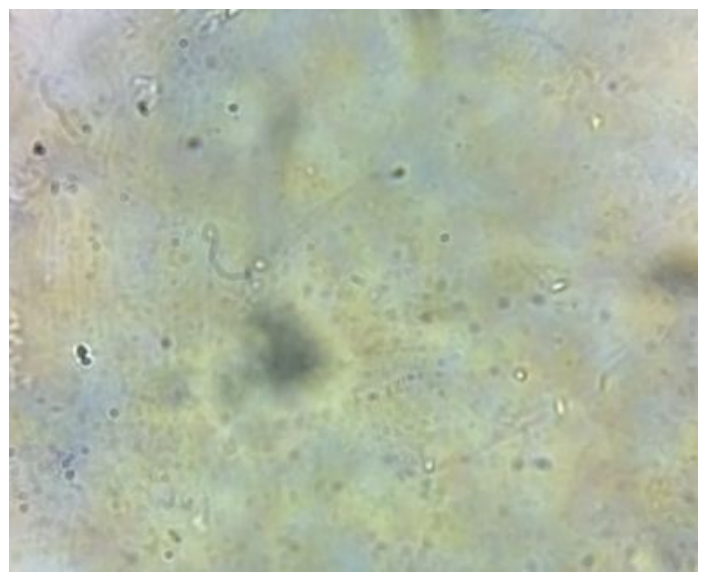

Molecular dynamics

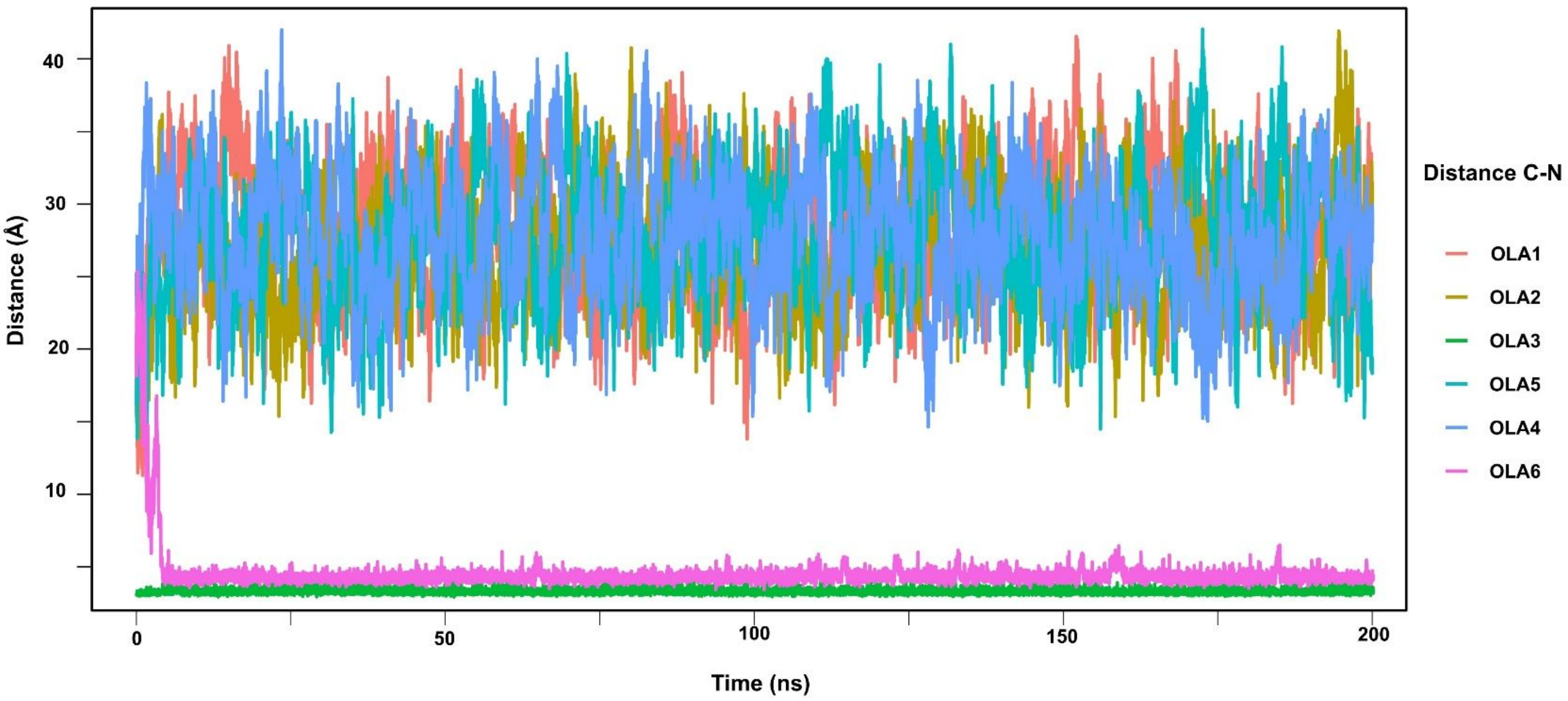

Figure S12. Distance between the carboxylate carbon atom of oleic acid (OA) and the nitrogen atom of the piperidine ring of donepezil (DP) as a function of simulation time. Each color-coded line corresponds to a distinct OA–DP complex.

Donepezil quantification

HPLC–UV analysis demonstrated the absence of interfering peaks originating from either skin components or formulation excipients at the retention time of donepezil (Figure S13), confirming the selectivity of the analytical method. Accordingly, the HPLC–UV method was suitable for reliable quantification of donepezil throughout the different stages of the study, including drug encapsulation in the nanoparticles, *in vitro* release experiments, and skin permeation assays.

A

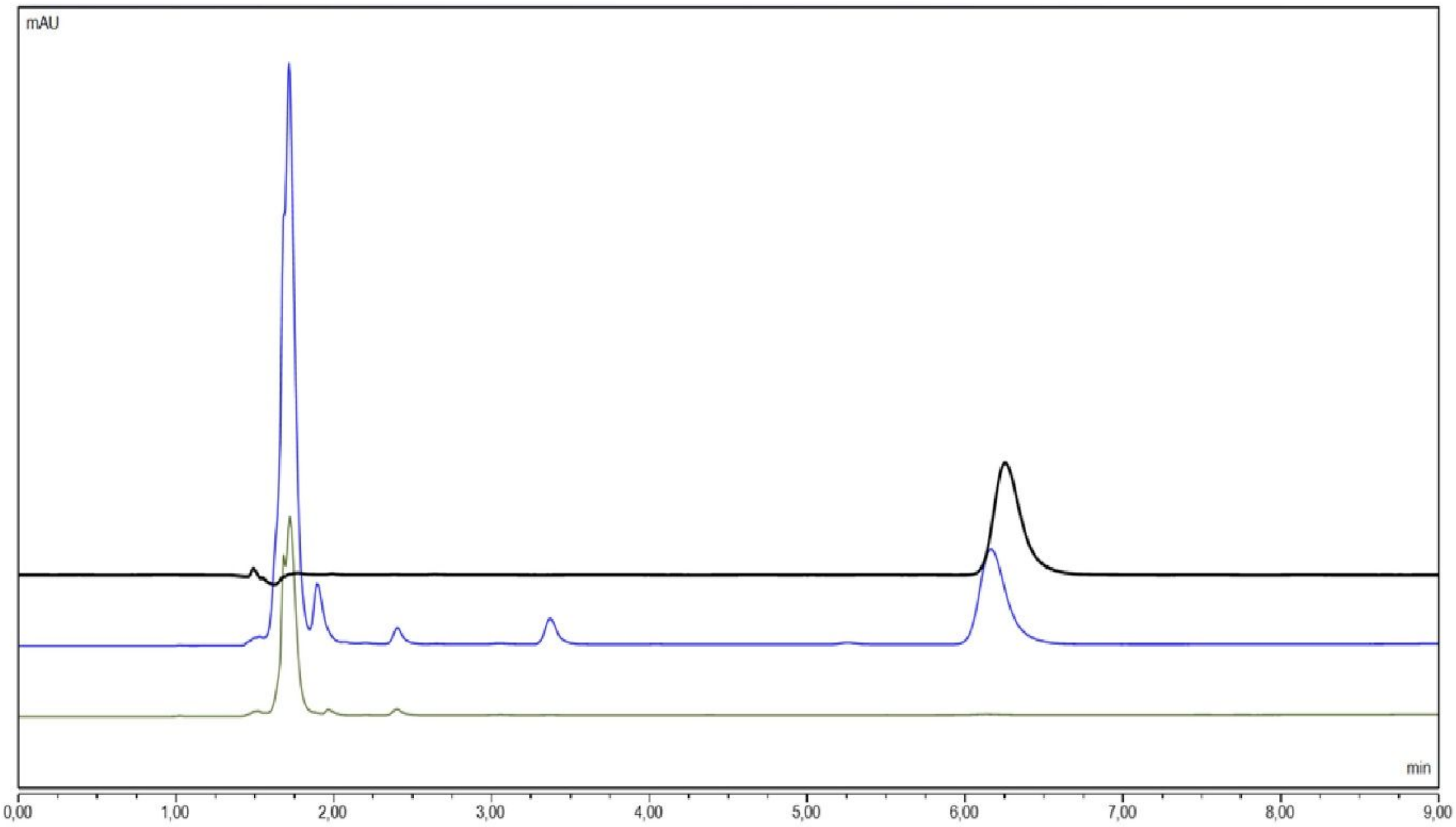

B

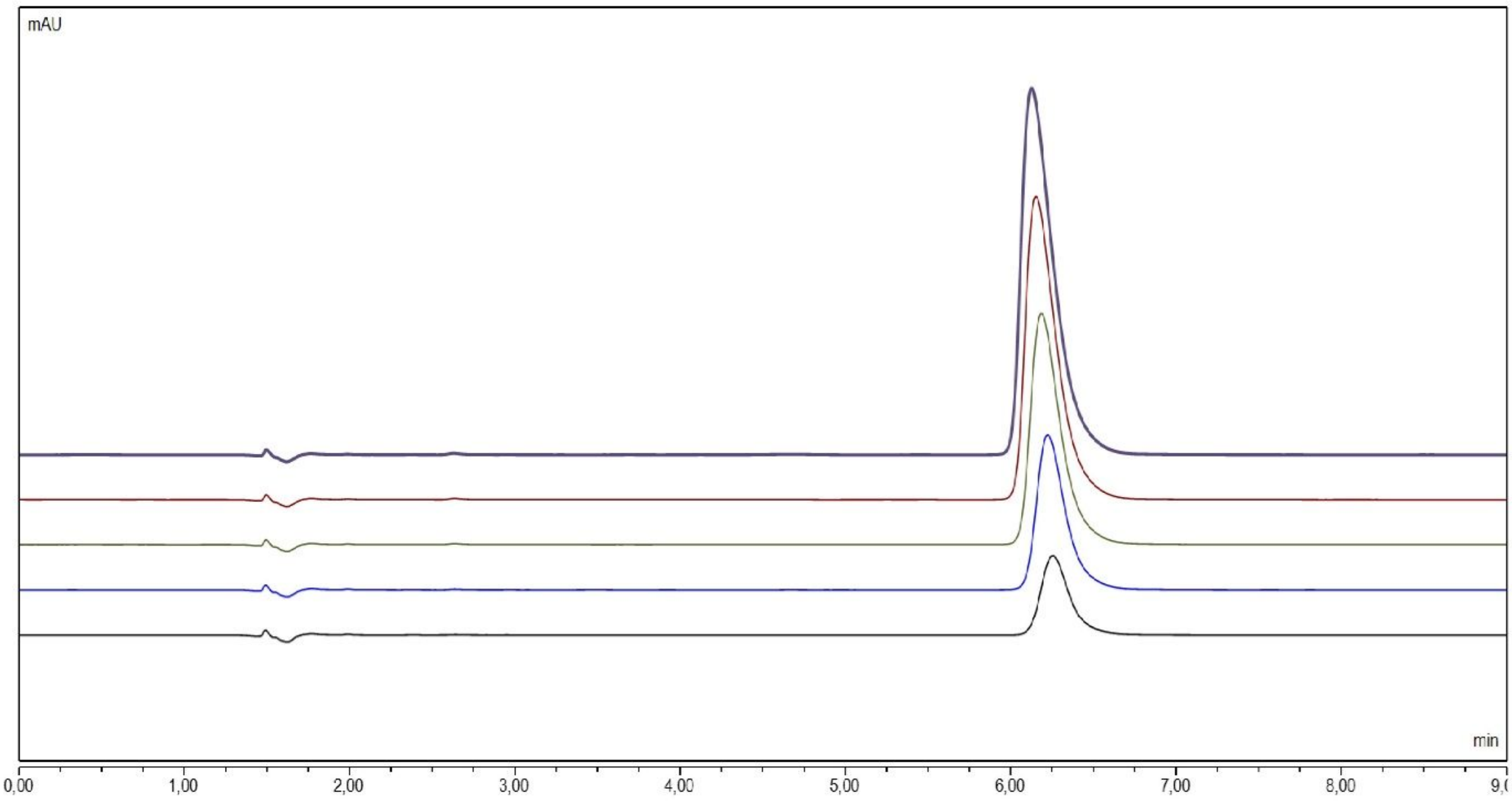

Figure S13. HPLC–UV analysis. (A) Overlay of chromatograms of the donepezil standard solution (black), sample solution obtained from the formulation in the skin permeation study (blue), and placebo formulation contaminated with skin (green). All samples were diluted in sodium acetate buffer (pH 4.5), which was used as the receptor medium in the in vitro release and skin permeation assays. (B) Overlay of chromatograms of donepezil standard solutions used for construction of the analytical calibration curve, prepared in the same sodium acetate buffer (pH 4.5).

System suitability was assessed based on the parameters listed in Table S1, with results consistent with literature recommendations for drug quantification [8]. Chromatographic parameters were calculated from donepezil peaks obtained during calibration curve evaluation, using sodium acetate buffer (pH 4.5) as the sample diluent.

Table S3. System suitability parameters for the analytical method used for donepezil quantification (n = 15)

Chromatographic Parameter	Results ± standard deviation	Specification
Retention time	6.10 ± 0.19 min	---
Capacity factor (k')	3.15 ± 0.13	$k' > 2.0$
Tailing factor (T)	1.71 ± 0.19	$T \leq 2.0$
Theoretical plates (N)	5754 ± 637	$N > 2000$

*According to Shabir (2003) [8].

Calibration curves were constructed using donepezil solutions over different concentration ranges, with all dilutions prepared in sodium acetate buffer (pH 4.5). Each concentration level was analyzed in triplicate, with replicates prepared from independently prepared donepezil stock solutions (1.0 mg/mL). For the concentration range of 10–50 µg/mL, the linear regression equation was $y = -0.4421\ x \pm 0.0395 + 0.6784 \pm 0.0011$, with a correlation coefficient (r) of 0.9998 ± 0.0001. For the range of 0.5–10 µg/mL, the equation was $y = 0.65328\ x \pm 0.00389 - 0.03872 \pm 0.00883$ ($r = 0.9998 \pm 0.0001$). For the lowest concentration range (0.2–1.0 µg/mL), the regression equation was $y = 0.64002\ x \pm 0.02090 - 0.01800 \pm 0.01208$, with $r = 0.9979 \pm 0.0009$. For all calibration curves, homoscedasticity was confirmed by the Breusch–Pagan test, with $p > 0.05$.

Atomic force microscopy

The atomic force microscopy (AFM) images were obtained from samples prepared by spreading 5 µL of the liquid crystalline nanodispersion-2 (LCN-2) onto freshly cleaved mica substrates (1.0 × 1.0 cm), followed by drying under a nitrogen stream for 30 s. All AFM images were processed using Gwyddion (version 2.67, Czech

Metrology Institute, 2024), an open-source software, which included leveling, profiling, and 3D rendering. Imaging in tapping mode proved difficult due to the strong adhesive characteristics of the sample (Figure S14A), resulting in intermittent sticking of the material to the AFM cantilever. Particle diameters were measured at half-height in the topographic images. The particle sizes determined by AFM (~50 nm) were smaller than the hydrodynamic diameters obtained by dynamic light scattering (approximately ~180 nm), which may be related to the presence of a polymer corona that provides steric stabilization in the aqueous medium.

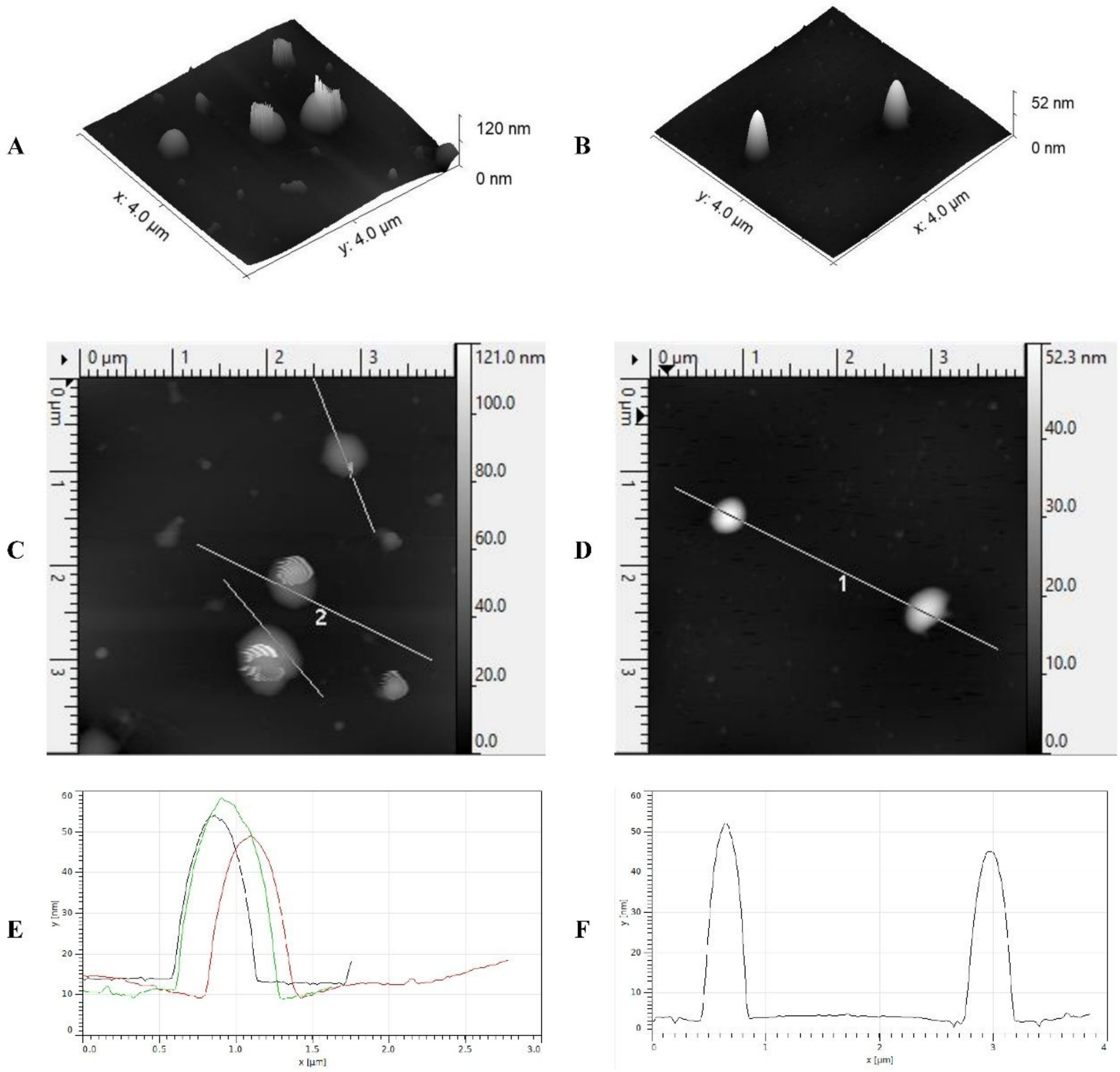

Figure S14. Atomic force microscopy images (4 µm × 4 µm fields): **A–B)** Topographical images of LCN-2; C–D) Positions used for half-height diameter measurements; **E–F)** Half-height diameter values.

References

(1) Asiri, Y. A.; Mostafa, G. A. E. Donepezil. In *Profiles of Drug Substances, Excipients and Related Methodology*; Brittain, H. G., Ed.; Academic Press Inc., 2010; Vol. 35, pp 117–150. https://doi.org/10.1016/S1871-5125(10)35003-5.

(2) G. F. Gomides, T.; C. Marques, V.; P. Silva, P.; R. R. Fernandes, G.; F. Jammal, N.; S. Marques, F.; P. M. Barboza, A.; M. A. Vieira, P.; de Souza, J.; de Souza Filho, J. D.; L. M. Ruela, A. The Role of Intermolecular Interactions between Donepezil and Methacrylic Acid in the Synthesis of Crosslinked Polymer Microgels for Drug Delivery. *J. Braz. Chem. Soc.* **2025**, 1–16. https://doi.org/10.21577/0103-5053.20250102.

(3) Nieva-Echevarría, B.; Goicoechea, E.; Manzanos, M. J.; Guillén, M. D. A Method Based on 1H NMR Spectral Data Useful to Evaluate the Hydrolysis Level in Complex Lipid Mixtures. *Food Res. Int.* **2014**, *66*, 379–387. https://doi.org/10.1016/j.foodres.2014.09.031.

(4) Aursand, M.; Grasdalen, H. Interpretation of the 13C-NMR Spectra of Omega-3 Fatty Acids and Lipid Extracted from the White Muscle of Atlantic Salmon (Salmo Salar). *Chem. Phys. Lipids* **1992**, *62* (3), 239–251. https://doi.org/10.1016/0009-3084(92)90061-S.

(5) Gusntone, F. D. 13C-NMR Studies of Mono-, Di-and Tri-Acylglycerols Leading to Qualitative and Semiquantitative Information about Mixtures of These Glycerol Esters. *Chem. Phys. Lipids* **1991**, *58* (3), 219–224. https://doi.org/10.1016/0009-3084(91)90095-S.

(6) Lankhorst, P. P.; Chang, A. *Modern Magnetic Resonance*; Webb, G. A., Ed.; Springer International Publishing: Cham, 2016. https://doi.org/10.1007/978-3-319-28275-6.

(7) Moreira, D. N.; Fresno, N.; Pérez-Fernández, R.; Frizzo, C. P.; Goya, P.; Marco, C.; Martins, M. A. P.; Elguero, J. Brønsted Acid–Base Pairs of Drugs as Dual Ionic Liquids: NMR Ionicity Studies. *Tetrahedron* **2015**, *71* (4), 676–685. https://doi.org/10.1016/j.tet.2014.12.003.

(8) Shabir, G. A. Validation of High-Performance Liquid Chromatography Methods for Pharmaceutical Analysis. *J. Chromatogr. A* **2003**, *987* (1–2), 57–66. https://doi.org/10.1016/S0021-9673(02)01536-4.
